# Dysfunction of the intestinal microbiome in inflammatory bowel disease and treatment

**DOI:** 10.1186/gb-2012-13-9-r79

**Published:** 2012-09-26

**Authors:** Xochitl C Morgan, Timothy L Tickle, Harry Sokol, Dirk Gevers, Kathryn L Devaney, Doyle V Ward, Joshua A Reyes, Samir A Shah, Neal LeLeiko, Scott B Snapper, Athos Bousvaros, Joshua Korzenik, Bruce E Sands, Ramnik J Xavier, Curtis Huttenhower

**Affiliations:** 1 Department of Biostatistics, Harvard School of Public Health, Boston, MA 02115, USA; 2 Broad Institute of Massachusetts Institute of Technology and Harvard University, Cambridge, MA 02142, USA; 3 Gastrointestinal Unit and Center for the Study of Inflammatory Bowel Disease, Massachusetts General Hospital, Harvard Medical School, Boston, MA 02114, USA; 4 Center for Computational and Integrative Biology, Massachusetts General Hospital, Harvard Medical School, Boston, MA 02114, USA; 5 Current address: Department of Gastroenterology, AP-HP, Hôpital Saint-Antoine and UPMC University of Paris, Paris, 75012, France; 6 Division of Pediatric Gastroenterology, Hasbro Children's Hospital, The Warren Alpert School of Medicine at Brown University, Providence, RI 02903, USA; 7 Gastrointestinal Unit, Children's Hospital and Brigham and Women's Hospital, Harvard Medical School, Boston, MA 02115, USA; 8 Department of Gastroenterology, Mount Sinai School of Medicine, New York, NY 10029, USA

## Abstract

**Background:**

The inflammatory bowel diseases (IBD) Crohn's disease and ulcerative colitis result from alterations in intestinal microbes and the immune system. However, the precise dysfunctions of microbial metabolism in the gastrointestinal microbiome during IBD remain unclear. We analyzed the microbiota of intestinal biopsies and stool samples from 231 IBD and healthy subjects by 16S gene pyrosequencing and followed up a subset using shotgun metagenomics. Gene and pathway composition were assessed, based on 16S data from phylogenetically-related reference genomes, and associated using sparse multivariate linear modeling with medications, environmental factors, and IBD status.

**Results:**

Firmicutes and Enterobacteriaceae abundances were associated with disease status as expected, but also with treatment and subject characteristics. Microbial function, though, was more consistently perturbed than composition, with 12% of analyzed pathways changed compared with 2% of genera. We identified major shifts in oxidative stress pathways, as well as decreased carbohydrate metabolism and amino acid biosynthesis in favor of nutrient transport and uptake. The microbiome of ileal Crohn's disease was notable for increases in virulence and secretion pathways.

**Conclusions:**

This inferred functional metagenomic information provides the first insights into community-wide microbial processes and pathways that underpin IBD pathogenesis.

## Background

Inflammatory bowel disease (IBD), a chronic and relapsing inflammatory condition of the gastrointestinal (GI) tract, is intimately linked to the microbial communities of the human gut. Although it is now widely accepted that IBD results from altered interactions between gut microbes and the intestinal immune system, the precise nature of the intestinal microbiota dysfunction in IBD remains to be elucidated [1]. IBD further includes two main subtypes, ulcerative colitis (UC) and Crohn's disease (CD), which each include distinct microbial perturbations and tissue localizations. The former is confined to the colon, while the latter may affect any part of the digestive tract, with unclear implications for microbial involvement or causality [2]. In particular, the microbial mechanisms and metabolism underlying the role of the GI microbiome in IBD onset and its alteration in the course of active treatment and recovery are still unknown.

In the last decade, advances in DNA sequencing have allowed exploration of the 40% of the gut microbiome that is still uncultured [3], setting the stage for investigation of the IBD microbiome. The GI microbiome of healthy humans is dominated by four major bacterial phyla: Firmicutes, Bacteroidetes, and to a lesser degree Proteobacteria and Actinobacteria [4,5]. Many studies have observed imbalances or dysbioses in the GI microbiomes of IBD patients [6-13]; in both CD and UC patients, there is decreased biodiversity, a lower proportion of Firmicutes, and an increase in Gammaproteobacteria [14]. In CD, proportions of the Clostridia are altered: the *Roseburia *and *Faecalibacterium *genera of the Lachnospiracae and Ruminococcaceae families are decreased, whereas *Ruminococcus gnavus *increases [15-17]. Specific features of UC-associated dysbiosis are less described, although increased sulfate-reducing Deltaproteobacteria have been reported [18,19]. These studies have described typical changes in composition of the IBD gut community, but the functional roles of these organisms - or of the entirety of a dysbiotic community - remain less clear.

The normal gut microbiome exhibits tremendous functional diversity encoded by a collection of bacterial genes numbering more than 100 times the human gene set [4,20]. Thus, the genomic potential of the human microbiome is far greater than that of its host, and treatments, diets, or medications that affect the host will also likely affect the microbiome. A primary example of the importance of the microbiome to host health is in the digestion of dietary fiber, which is used by the microbiota of the lower GI tract as their main source of energy [21]. Fibrolytic bacteria degrade polysaccharides into smaller carbohydrates, which are then fermented into short-chain fatty acids (SCFAs) such as acetate, propionate, and butyrate. Butyrate in particular is a major source of energy for colonocytes, but all three of these have demonstrated immunomodulatory properties [22-27]. In addition to these metabolic functions, many genetic studies in IBD have highlighted the central role of host-microbe interactions in IBD pathogenesis [1,28-30]. Specific host pathways linked to microbial response in IBD include T-cell activation, the IL-23/T helper 17 pathway, autophagy [31], and Paneth cell function [32]. Together, these results support the centrality of host-microbiota crosstalk for gut homeostasis and in turn the role of dysfunctional crosstalk between the host and GI microbiome in IBD.

Little work has yet bridged the gap between IBD pathogenesis in a human host, individual microbes, and alterations in metabolism of the GI microbial community in IBD. Few studies of the IBD gut microbiome have investigated microbiome function [33], and these have not systematically accounted for the influences of treatments and environmental factors. We have thus analyzed the GI microbiomes of 121 CD patients, 75 UC patients, and 27 healthy controls using a novel multivariate metagenomic analysis pipeline specifically accounting for environmental factors (including treatment, age, and tobacco use). In addition to assessing microbiome composition, we have analyzed the inferred metagenome as determined from phylogenetically-associated reference genomes, including metabolic modules and pathways also associated with disease status and with environmental factors such as medications and smoking. Not only were these GI microbiomes characterized by shifts in bacterial populations during disease as previously described, but these dysbioses were highly functionally coordinated. Cross-species enrichments included mucin metabolism and redox tolerance by means of glutathione transport, cysteine biosynthesis, and riboflavin metabolism. Conversely, processes linked broadly to clades IV and XIVa Clostridia were depleted, particularly short chain fatty acid production. Dysbioses in IBD are correspondingly not simply structural changes in the gut microbiota, but are instead associated with major impairments in many fundamental microbial metabolic functions with potential impact on the host.

## Results

In order to measure compositional and functional differences between the gut microbiota of healthy and IBD-affected individuals, 231 fecal and biopsy samples were collected from the Ocean State Crohn's and Colitis Area Registry (OSCCAR) and the Prospective Registry in IBD Study at MGH (PRISM) database. OSCCAR is a state-based, prospective inception IBD cohort, and PRISM is a referral center-based, prospective IBD cohort (see Materials and methods). The samples comprised 136 fecal specimens and 95 colon or small intestinal biopsies, originating from a cross-section of 121 CD patients, 75 UC patients, 27 healthy controls, and 8 indeterminate (Table 1). In addition to general information such as gender and age, data regarding disease characteristics (topography, disease activity as measured by the Harvey-Bradshaw Index (HBI) and the Simple Colitis Activity Index), treatment (antibiotics, corticosteroids, mesalamine, immunosuppressant), and environmental exposure (tobacco use) were collected from each subject and analyzed. DNA was extracted from fecal samples and biopsies, and the 16S rRNA gene was amplified and sequenced using 454 technology. The resulting sequences were then processed using a specific *in silico *pipeline for sequence cleaning and phylotype assignment (see Materials and methods). At the end of this process, the average sequencing depth was 2,860 reads per sample. These data were first validated by comparison with previous work, recapitulating previously observed changes in microbial community composition during IBD and attributing several to host treatment or environment. They were subsequently associated with reference genomes in order to discover disease-associated modulations of microbial function and metabolism. A subset of 11 samples (7 healthy, 4 CD) were subjected to whole-genome shotgun sequencing using the Illumina MiSeq platform at an average depth of 119 meganucleotides per sample in order to confirm these functional inferences.

**Table 1:** Characteristics of patients in this study

	CD	UC	HS	Indeterminate
n	121	75	27	8
Female gender (n)	59.5% (72)	49.3% (37)	55.6% (15)	62.5% (5)
Age (lower 95%-upper 95%)	37.3 (34.3-40.3)	41.1 (37.4-44.9)	35.1 (29.1-41.2)	26.9 (13.4-40.3)
**Smoker (n)**				
Never	63.6% (77)	57.3% (43)	85.2% (23)	75.0% (6)
Previously	24.8% (30)	40% (30)	11.1% (3)	12.5% (1)
Current	10.7% (13)	2.7% (2)	0% (0)	12.5% (1)
Unknown	0.8% (1)	0% (0)	3.7% (1)	0% (0)
**Sample**				
Stool (n)	51.2% (62)	64% (48)	66.7% (18)	100% (8)
Biopsy (n)	48.8% (59)	36% (27)	33.3% (9)	0% (0)
**Disease**				
Active disease (n)^a^	26.4% (32)	29.3% (22)	0% (0)	0% (0)
Ileal (n)	35.5% (43)	NA	NA	NA
**Treatment**				
Mesalamine (n)	55.4% (67)	77.3% (58)	0% (0)	75.0% (6)
Steroids (n)	31.4% (38)	37.3% (28)	0% (0)	50% (4)
Immunosuppressant (n)^b^	38.8% (47)	16% (12)	0% (0)	0% (0)
Antibiotics (n)	12.4% (15)	13.3% (10)	0% (0)	12.5% (1)

^a^Active disease defined by a Harvey-Bradshaw Index (HBI) ≥ 5 or Pediatric Crohn's Disease Activity Index (pCDAI) ≥ 10 for Crohn's disease (CD), and Simple Clinical Colitis Activity Index ≥ 5 or Pediatric Ulcerative Colitis Activity Index (pUCAI) ≥ 10 for ulcerative colitis (UC). ^b^Immunosuppressant treatments include thiopurines, methotrexate, and anti-tumor necrosis factor-α antibody. HS, healthy subjects; NA, not applicable.

### Assessing significant covariation of microbiome structure with host IBD status, treatment, and environment

**Figure 1:**
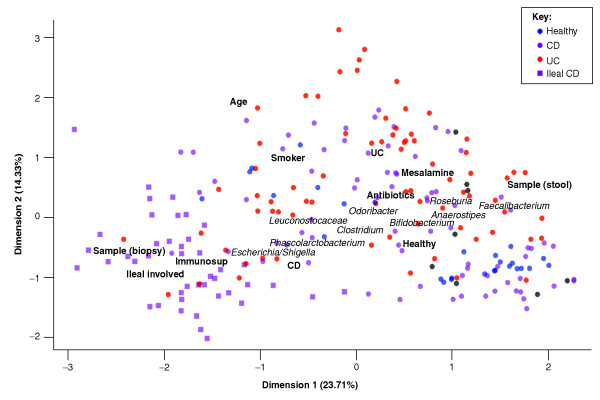
**Covariation of microbial community structure in IBD with treatment, environment, biometrics, and disease subtype**. Fecal and biopsy samples from 228 IBD patients and healthy controls are plotted as squares (ileal CD) or circles (not ileal involved) and colored by disease status. Axes show the first two components of overall variation as determined by multiple factor analysis (see Materials and methods). Covariation in the presence of clinical factors (bold) and in microbial taxa (italic) is shown. Sample origin (biopsy versus stool) is the single most influential factor in determining microbial community structure, accompanied by host age, treatment types, and disease (particularly ileal CD).

We used a sparse multivariate statistical approach to relate disease phenotype to microbiome structure and function while accounting for potential correlates and confounding factors such as treatment or smoking. Metadata features potentially associated with each clade were first selected using boosting, and the significance of these associations was then assessed using a multivariate linear model with false discovery rate correction (see Materials and methods). We first investigated the resulting association of microbial clades with IBD and with features of our cohorts, testing all available metadata and clades from the genus to phylum levels. Ordination of overall relationships among samples and host status revealed several major combinations of environmental factors that co-varied with the microbiome (Figure 1; Additional file 1). For example, UC covaried in this population with mesalamine treatment, whereas CD patients were more often assessed by biopsy, treated with immunosuppressants, and enriched for *Escherichia*. Similarity among microbiome compositions in disease subtypes reflects those previously observed [34,35], with ileal CD (iCD) representing a strong outgroup, UC a generally less-extreme microbial phenotype (less dissimilar from healthy subjects), and non-iCD a broad distribution of microbiome configurations.

An important consideration that informed the remainder of our analysis, and which is often overlooked in studies of the microbiome, was the consistent covariation among disease status, aspects of subject environment, and microbiome structure. For example, the factor most associated with changes in microbiome composition was not disease but whether the sample origin was stool or biopsy. Biopsy location induced minor changes in microbiome composition (Additional files 2, 3 and 4) relative to the extreme differences between stool and biopsy communities, in agreement with previous studies [36,37]. In this cohort, iCD was always represented by biopsy, whereas 18.4% of non-iCD and 36% of UC samples were biopsies. iCD was also associated with greater likelihood of immunosuppressant treatment: iCD, non-iCD, and UC patients were treated by immunosuppressants in 74.4%, 19.2% and 16% of samples, respectively. In contrast, non-iCD and UC cases were more likely to be treated with mesalamine or antibiotics: mesalamine was used for 30.2% of iCD samples, 69.2% of non-iCD samples, and 77.3% of UC samples, while antibiotics were used in 2.3% of iCD, 17.9% of non-iCD, and 13.3% of UC samples. These associations lead to a range of non-independent covariates. Although disease activity may influence microbiome composition, after adjusting for the other factors, it was not independently associated with a specific shift in the microbiome composition in our analysis, and there were no significant (*P *< 0.01) associations between microbiome composition and gender (Additional file 5).

The second largely independent factor influencing microbiome composition was age, itself negatively associated with smoking (Figure 1; Additional file 1). Twenty-four (10.4%) of the available subjects were less than 18 years of age and 26 were 60 years or older. Aging is associated with continual changes in the microbiome, primarily a gradual decrease in *Bifidobacterium *as observed here (Additional file 6) and by others [38,39]. After observing these overall patterns of covariation among disease, treatment, environment, and gut microbiome composition, we continued our analysis only after assessing the significance of microbiome-disease associations in a multivariate manner to account for host environment and treatment.

### Microbial clades differentially abundant specifically in IBD include Roseburia, the Ruminococcaceae, and the Enterobacteriaceae

**Figure 2:**
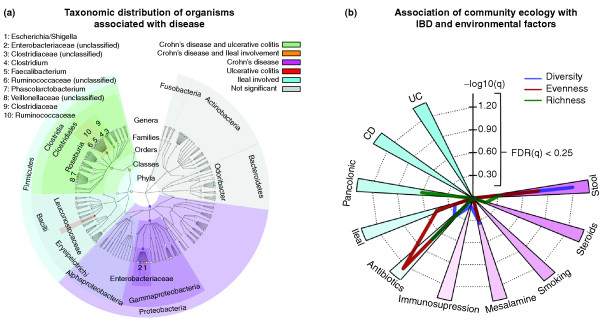
**Significant associations of microbial clade abundance and community ecology with IBD and treatment**. **(a) **Taxonomic distribution of clades significant to disease and ileal involvement. Abundant clades not significantly associated with IBD are annotated in gray for context (top 90th percentile of at least 10% of samples and including 5+ genera). Node (non-associated clade) sizes are proportional to the log of the clade's average abundance. **(b) **Significance of association of sample ecology with disease (CD/UC, ileal/pancolonic), treatment (antibiotics, immunosuppression, mesalamine, steroids), and environment (smoking, stool/biopsy sample origin). Diversity (Simpson's index), evenness (Pielou's index), and richness (Chao1) were calculated for each community (see Materials and methods). False discovery rate q-values are -log_10 _transformed for visualization, such that values > 0.60 correspond to q < 0.25. Antibiotic treatment is strongly associated with reduced diversity, and stool samples with increased diversity relative to biopsies.

After adjusting for these covariates, we determined microbial clades differing significantly in abundance between healthy and IBD subjects (Figure 2a; Additional file 1). This considered age, smoking, and treatment factors (immunosuppressant, corticosteroids, mesalamine, antibiotics), as well as disease activity at sampling and sample type (stool or biopsy). Two genus-level phylotypes, *Roseburia *and *Phascolarctobacterium*, were significantly reduced in both UC and CD, while *Clostridium *increased, all with false discovery rate q < 0.2. *Roseburia *is a clade XIVa Clostridia and thus associated with anti-inflammatory regulatory T cell production in the gut [40]. Cultured *Roseburia *have been described as acetate utilizers and butyrate producers [41], while cultured *Phascolarctobacterium *are exclusively succinate consumers, and produce propionate when co-cultured with *Paraprevotella *[42]. Thus, an IBD-associated decrease in *Roseburia *and *Phascolarctobacterium *may reflect a decrease in butyrate and propionate production.

The Ruminococcaceae, which are acetate producers [43], were decreased in CD, while the Leuconostocaceae, which produce acetate and lactate [44], were decreased in UC. The only major clade with a significant increase in abundance specific to CD was the Enterobacteriaceae, specifically *Escherichia/Shigella*. This family has been previously implicated in intestinal inflammation [6,45-47].

### Crohn's disease with ileal involvement presents a distinct microbiome phenotype including reduced Faecalibacterium, and Odoribacter is reduced both in iCD and in pancolonic UC

In CD patients with ileal involvement, sequences of the Ruminococcaceae family and of *Faecalibacterium *in particular were dramatically reduced compared to other subjects (Figure 2a), confirming previous studies [48,49]. *Faecalibacterium prausnitzii*, the only cultured representative of *Faecalibacterium*, is able to metabolize both diet-derived polysaccharides and host-derived substrates such as N-acetyl glucosamine from intestinal mucus [50]. It is also a major butyrate producer and exhibits anti-inflammatory effects in a colitis setting [51]. The Ruminococcaceae represent the first step of microbiome-linked carbohydrate metabolism, as they degrade several types of polysaccharides in the lower GI tract, including starch, cellulose, and xylan [21]. The *Roseburia *genus, which is significantly reduced in all IBD patients (including iCD), and the Ruminococcaceae are further functionally connected in that the latter consume hydrogen and produce acetate that can be utilized by *Roseburia *to produce butyrate [41,43]. Consistent reductions in all of these clades may thus have functional consequences on the ability of the host to repair the epithelium and to regulate inflammation.

The genera *Escherichia/Shigella *(indistinguishable as a 16S-based phylotype) were particularly highly enriched in iCD (q < 0.2; Additional file 1) above their general overabundance in CD patients. Lipopolysaccharide produced by Gram-negative bacteria such as *Escherichia coli *is a canonical microbe-associated molecular pattern, known to activate toll-like receptor 4 (TLR4) signaling [52] and thus trigger inflammatory cascades. TLR4 expression is highly up-regulated in the intestinal epithelium of IBD patients [53], and mutations in TLR4 are associated with both CD and UC [54]. Previous culture-based studies have found that *E. coli*, specifically *E. coli *exhibiting pathogen-like behaviors such as adhesion and invasiveness [55], are more frequently cultured from iCD biopsies, and culture-independent studies have found an enrichment in *E. coli *that contain virulence-associated genes in iCD [6]. This suggests that CD-involved ileum is a favorable milieu for establishment of *E. coli *with pathobiont features, which may have implications for IBD exacerbations and its chronicity. An inflamed ileum may furnish a specialized niche permissive for microbes with enhanced fitness in inflamed conditions.

The most severe form of UC is pancolitis, in which UC affects the entire colon; this condition is associated with greatly increased risk of colon cancer [56]. Patients with pancolitis did not harbor a clear specificity in their dysbiosis. However, both these patients and iCD patients had a reduced abundance of the *Odoribacter *genus, which belongs to the Porphyromonadaceae family and to the Bacteroidetes phylum. As *Odoribacter splanchnus *is a known producer of acetate, propionate, and butyrate [57], decreased *Odoribacter *may affect host inflammation via reduced SCFA availability.

### Microbiome composition is also strongly associated with subject age, treatment, smoking, and sample biogeography

**Figure 3:**
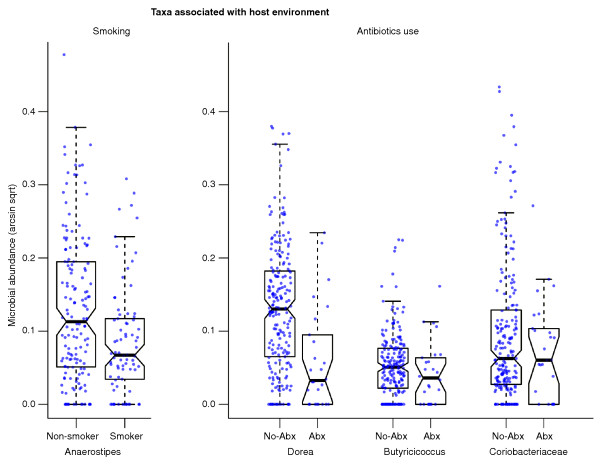
**Select microbial clades significantly linked to host environment and treatment**. Anaerostipes decreased significantly in the gut communities of smokers, and *Dorea, Butyricicoccus*, and Coriobacteriaceae were among the taxa most reduced in patients receiving antibiotics (Abx). These associations were significant even in a multivariate model accounting for sample biogeography and disease status. Sqrt, square root.

In the process of identifying microbiome perturbations specific to IBD, our multivariate model simultaneously analyzed the surprisingly diverse effects of environmental and treatment factors on GI microbial communities (see selection in Figure 3; complete data in Additional file 1). We observed a significant correlation between increasing age and decreasing *Bifidobacterium *(Additional file 6). The Firmicutes phylum also significantly decreased while Bacteroides increased with age in this cohort (Additional file 1); this agrees with previous studies [38,39] and potentially reflects dietary or body mass-related changes with increasing age, which were not directly measured in these subjects, or host metabolism modifications [58].

Critical to determining causality in links between IBD and the gut microbiome, IBD treatments were also associated with alterations in microbiome composition. Mesalamine (5-aminosalicylic acid) is a bowel-specific aminosalicylate drug. Although its exact mode of action is unknown, it is thought to act as an antioxidant and to decrease intestinal inflammation, in part by peroxisome proliferator-activated receptor-γ (PPARγ) activation and inhibition of NFκB and pro-inflammatory eicosanoid production. Here, its use was linked to strong reductions in *Escherichia/Shigella *(> 100% of average abundance, q < 0.04; Additional file 7), in agreement with a recent study [59]. Both 5-aminosalicylic acid and immunosuppressant treatment were associated with modest increases in *Enterococcus*, the only genus perturbed in immunosuppressant-treated patients with low false discovery rate (also > 100% of average abundance, q < 0.09).

Antibiotics were among the strongest factors associated with a reduction in ecological diversity (Figure 2b). Many individual clades were greatly reduced or nearly absent after administration of antibiotics, including the *Collinsella, Dorea, Butyricicoccus, Subdoligranulum*, and *Acetivibrio *(all q < 0.2; Additional file 1). These genera are predominantly from the Clostridiales order, Gram-positive and anaerobic bacteria that are targeted by the antibiotics commonly used in IBD, such as ciprofloxacin and metronidazole.

Smoking is likely the best-known environmental factor that impacts IBD [60]. It is associated with increased risk of CD and is conversely protective towards developing UC [61]. The only common organism to which tobacco usage was linked in these individuals was *Anaerostipes *(Firmicutes phylum), which decreased (> 60% average abundance, q < 0.15; Figure 3) in current or former tobacco users, beyond any change due solely to smokers' higher average age. The *Anaerostipes *genera can utilize lactate to produce butyrate [62], which is beneficial to colonic health.

Finally, as previously mentioned, samples of the stool as opposed to mucosal biopsies differed strongly in microbiome composition (Additional file 2). More than 70 clades were significantly over- or under-enriched in stool samples relative to biopsies at q < 0.2. This effect extended to entire phyla, as the Firmicutes were approximately twofold more abundant in stool (Additional file 1). Microbial habitat dictates the composition of microbial communities [36]; in the GI tract, this has been suggested to occur on biogeographical scales of intestinal regions [37,63] or even millimeters apart [64,65], and luminal/mucosal differences may be further perturbed by bowel preparation prior to colonoscopy [66]. The data did not suggest that the luminal and mucosal communities were independent; rather, all 14 clades significantly associated with IBD retained the same trend when stratified by sample origin (Additional file 8). The fecal microbiome appeared to convey a consistent but numerically transformed function of mucosal communities, both of which shifted in composition in association with host environment, treatment, and disease.

In a closer analysis of intestinal biogeography as reflected by biopsies drawn from distinct regions, differences in most clades were modest and correlated largely with previously described changes in pH (Additional files 2, 3 and 9) [67]. The clades with the largest regional changes included the *Roseburia *and Ruminococcaceae, with lower abundance in the low-pH terminal ileum, transverse, and right colon; *Alistipes*, following a similar pattern; and the *Fusobacteria *and Enterobacteriaceae, with an opposite pattern of somewhat increased abundance in the ileum and right colon. Particularly as the former have also been associated with the colorectal cancer microenvironment in previous work [68,69], it is of note that these variations in the microbiota with respect to biogeography and pH are similar to those we observed with respect to IBD and potentially redox status as detailed below.

### The metagenomic abundances of microbial metabolic pathways are more consistently perturbed in IBD than are organismal abundances

**Figure 4:**
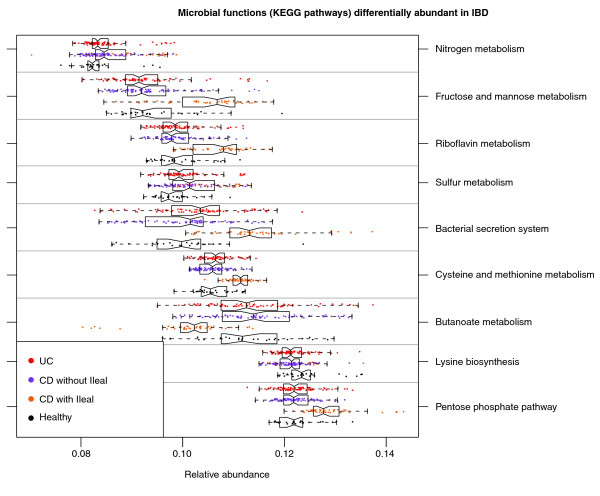
**Microbial metabolic pathways with significantly altered abundances in the gut communities of IBD patients**. Abundance of KEGG metabolic pathways in microbiome samples is colored by disease state and, when significant, stratified by ileal involvement. Basic metabolism (for example, most amino acid biosynthesis) and SCFA production were reduced in abundance in disease, while biosynthesis and transport of compounds advantageous for oxidative stress (for example, sulfur, cysteine, riboflavin) and adherence/pathogenesis (for example, secretion) were increased.

**Figure 5:**
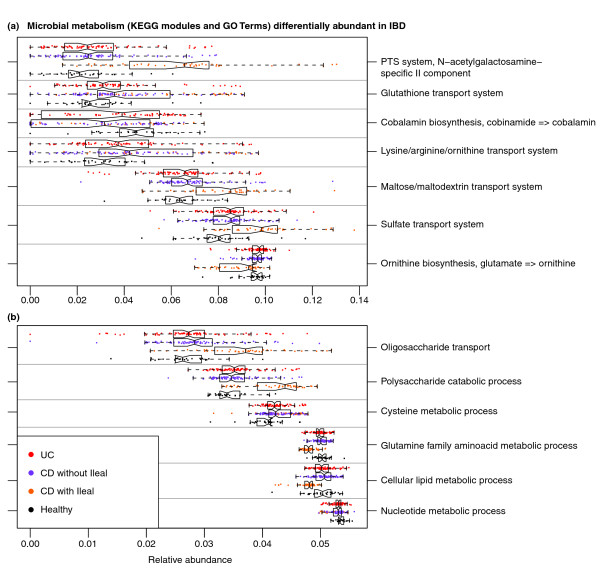
**Small metabolic modules and biological processes with significantly altered abundances in the IBD microbiome**. **(a, b) **Small (typically 5 to 20 gene) KEGG modules (a) and independently defined biological processes from the Gene Ontology (b) were assessed for significant association with disease and ileal involvement as in Figure 4. Metabolism related to oxidative stress (for example, glutathione and sulfate transport) and for pathobiont-like auxotrophy (for example, N-acetylgalactosamine and amino acid uptake) is increased, while several basic biosynthetic processes are less abundant.

We continued our analysis by combining community composition with over 1,200 annotated genomes from the Kyoto Encyclopedia of Genes and Genomes (KEGG) catalog [70]. The genes annotated within each available reference genome were used to provide an approximate gene catalog for each community (see Materials and methods), which we reconstructed into metabolic pathways (Figure 4) and smaller modules and biological processes (Figure 5; Additional file 10) as previously described [71]. Pathway, module, and process abundances were then associated with disease and host environment using the same sparse multivariate model with which microbial abundances were assessed (Additional files 11, 12 and 13).

Considering only the contrast between IBD (CD or UC) and healthy subjects, 24 of 200 (12%) total metabolic modules were differentially abundant at q < 0.2. This is in stark contrast to the microbial shifts discussed above, in which only 6 of 263 (2%) genus-level clades reached this significance threshold. Even in the absence of metagenomic or metatranscriptomic data and only leveraging the genes and pathways in reference genomes associated with these communities, changes in microbial function were more consistent than changes in community structure. This has been noted in environmental communities [72] and suggested with respect to obesity and other biometrics [73,74], but to date it has not been reported for disease-linked dysbioses or IBD.

We validated these functional shifts by shotgun metagenomic sequencing of the small subset of available samples with appropriate stool DNA, seven healthy controls and four CD patients (Additional file 14). These were sequenced to a shallow depth averaging 119 meganucleotides per sample of 150-nucleotide paired-end Illumina MiSeq reads, reducing our effective limit of detection but otherwise providing close agreement with inferred metabolic shifts in the IBD metagenome. Of the modules highlighted below and in Figure 5, one (cobalamin biosynthesis) fell below the limit of detection, and the remaining six retained the expected trend of over- or under-enrichment in Crohn's disease, as did additional processes detailed below, including glycolysis and bacterial secretion.

### Amino acid biosynthesis and carbohydrate metabolism are reduced in the IBD microbiome in favor of nutrient uptake

We observed that even basic GI microbiome metabolism was altered in both UC and CD. Amino acid metabolism showed major perturbation: genes for the metabolism and biosynthesis of nearly all amino acids (particularly histidine and lysine) decreased in abundance (Figure 4), while arginine, histidine, and lysine transport (Figure 5) gene abundance increased. In iCD we also observed a decrease in glutamine-related functional modules, which would lead to a lower amount of glutamate required for gamma-aminobutyric acid, ornithine, and arginine biosynthesis; abundance of all three of these modules also decreased. In marked contrast to the other amino acids, genes for metabolism of the sulfur-containing amino acid cysteine significantly increased in abundance, with even greater increase in iCD. This corresponded with an overrepresentation of genes related to sulfate transport in UC and CD (Figure 5), and in increase in sulfur and nitrogen metabolism in CD (Figure 4).

CD was associated with increased abundance of many genes related to carbohydrate transport (Figure 5). There were large increases in pentose phosphate pathway and fructose/mannose metabolism gene abundance in iCD (Figure 4), which were accompanied by increase in carbohydrate metabolism, but they were not significant in UC and CD. In addition, iCD showed increased abundance of transporter genes for glucose, hexoses, maltose, and mono-, di-, and oligosaccharides (Figure 5). We observed a decrease in both butanoate and propanoate metabolism in iCD (Figure 4), suggesting a potential decrease in SCFA production by the microbiome, possibly due to the observed decrease in *Roseburia *and *Faecalibacterium*.

We saw an increase in glutathione transport gene abundance in UC and CD (Figure 5) and an increase in glutathione metabolism gene abundance in UC. Glutathione is a tripeptide of cysteine and glutamate, synthesized by Proteobacteria and a few streptococci and enterococci [75], which allows bacteria to maintain homeostasis during oxidative or acid stress. Inflammatory cascades include production of highly reactive oxygen and nitrogen metabolites, which are greatly increased in active IBD [76]. Lamina propia monocytes also release homocysteine during inflammation, which further contributes to oxidative stress; IBD is associated with higher levels of both mucosal and serum homocysteine [77]. Thus, the increases in sulfate transport, cysteine metabolism, and glutathione metabolism may reflect a mechanism by which the gut microbiome addresses the oxidative stress caused by inflammation.

### Extreme functional shifts in iCD include changes in redox metabolism, enrichment of signaling/secretion, and suggest a 'pathobiont-like' invasive metagenome

CD with ileal involvement exhibited specific dysfunction at the module level. It was associated with an increase in several modules involved in glycolysis and carbohydrate transport and metabolism (Figure 5). Conversely, iCD exhibited lower abundance of genes involved in lipid metabolism and catabolism, confirming a major imbalance in energy metabolism. We observed a global decrease in nicotinamide, purine, and pyrimidine nucleotide biosynthesis modules in iCD, CD, and UC (Figure 5).

There was a decrease in vitamin biosynthesis associated with iCD, but increases in thiamine and particularly riboflavin metabolism modules (Figure 4). Interestingly, this pathway is fed by the pentose phosphate pathway, which was also overrepresented in iCD. Riboflavin is necessary for regenerating oxidized glutathione back to its reduced form, and is thus essential for pH and oxidative stress homeostasis, as is NADPH, a product of the pentose phosphate pathway. Metabolism of the sulfur-containing amino acids cysteine and methionine was increased in iCD, in marked contrast to the IBD-associated decreases in the non-sulfur-containing amino acids such as lysine and glutamine. As homocysteine is easily convertible to methionine, this may indicate a further mechanism of maintaining redox homeostasis. Alternatively, this may be connected to the iCD-specific increase in carbohydrate metabolism, as cysteine may be metabolized to pyruvate.

Finally, genes involved in pathogenesis processes, such as secretion systems and adherence/invasion, were overrepresented in iCD (Figure 4). For example, genes involved in the shigellosis pathway were more abundant in CD, and type II secretion genes were more abundant in iCD. Type II secretion is involved in the secretion of cell wall-degrading enzymes [78] and the secretion of toxins such as heat-labile enterotoxin, similar to cholera toxin [79]. These functions are typical of pathobiont adherent-invasive *E. coli*, which have been observed to increase in iCD in our own study and others [6,55]. This may be associated with tissue damage, either primarily as a result of toxin secretion, or secondarily as a result of stimulated cytokine production. This tissue destruction is a likely source of metabolites for microbial overgrowth, selecting for auxotrophic specialists able to thrive in this environment and resulting in the microbiome-wide loss of basic biosynthetic processes (Figures 4 and 5). This would in turn lead to further tissue breakdown, bacterial overgrowth, and community structural and functional dysbiosis.

## Discussion

The GI microbiome influences dietary energy extraction, immune system development, vitamin production, and drug metabolism, yet most molecular and metabolic functions of the bacteria of the GI microbiome are uncharacterized [20]. To gain insight into the functional consequences of IBD-associated dysbiosis, we used a novel approach pairing microbial community 16S gene sequence profiles with information from the closest available whole-genome sequences. This defined an inferred metagenome and thus complement of metabolic functional modules for each microbiome in this study. This allowed us to identify unique functional perturbations in the microbiomes of IBD patients. Interestingly, although we identified only nine changes in bacterial clades that associated with UC (of 350 total, 2.6%), we identified 21 statistically significant differences in functional pathways and metabolic modules (of 295, 7.1%); this pattern held for CD and iCD function as well. This underscores the fact that phylogenetically diverse changes in the composition of the GI microbiome can be functionally coordinated and lead to major modifications in the metabolic potential of the microbiota.

The microbial metabolic information available in this study represents only one step in the functional investigation of the IBD microbiota, as it is an accurate but approximate inference using prior knowledge of microbial genomes. The metagenomes inferred from our 16S data were supported by shotgun sequencing of a subset of samples, providing one confirmation that they were representative of community functional capability. As sequencing costs continue to fall, rich metagenomic data for dozens or hundreds of samples will further improve our ability to resolve species-level gene function in communities. Of course, a community expresses only a variable subset of its functional capability at any given time, in response to environmental stimuli. Thus, metatranscriptomic, proteomic, and metabolomic data will continue to add to our understanding of which of a community's potential functions are most strongly affecting the host during inflammatory disease.

**Figure 6:**
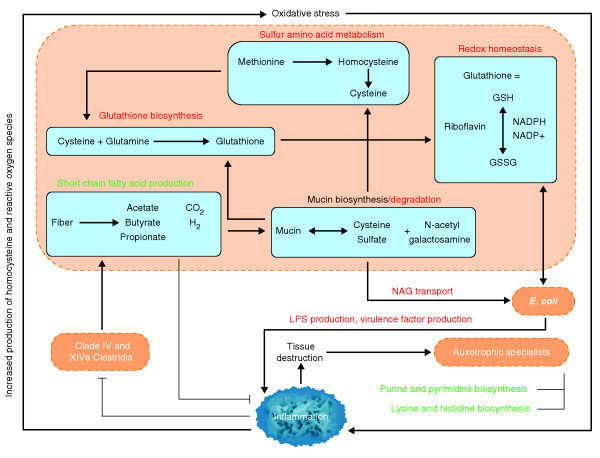
**Proposed metabolic roles of the gut microbiome in IBD**. Host-mediated processes (blue text) create an environment of oxidative stress in the intestine, which is more favorable to Enterobacteriaceae (increased abundance) than to clades IV and XIVa Clostridia (decreased abundance). This study's inferred IBD metagenomes include broadly increased oxidative metabolism, decreased SCFA production, and increased mucin degradation relative to healthy subjects. These processes all occur within microbes and rely on transport of small molecules to and from the lumen. The resulting tissue-destructive environment provides nutrients such as nucleotides and amino acids, which allow for increased growth of auxotrophic 'specialists'. Bacterial clades of interest are indicated in orange, bacterially mediated processes increased in IBD in red, and processes that decrease in green. Metabolic pathways differential in our IBD communities are contained in blue boxes. GSH and GSSG indicate reduced and oxidized forms of glutathione. LPS, lipopolysaccharide; NAG, N-acetyl galactosamine.

Combining shifts in functional module abundance with prior knowledge of these metabolic pathways provides fresh insight into microbiome dysfunction in IBD. Metabolism of the sulfur-containing amino acid cysteine was increased in both UC and CD. This was accompanied by increases in riboflavin metabolism, glutathione transporters, and the N-acetylgalactosamine phosphotransferase system. Mucin, which is rich in cysteine and glycosylated sugars, is abundant in the intestinal epithelium, and it is upregulated during inflammation. The increases in cysteine metabolism and N-acetylgalactosamine transporters may reflect a shift in the microbiome towards greater abundance of microbes that use mucin as a primary energy source (Figure 6). This functionality suggests activity at the mucosa and this may be problematic for a damaged IBD epithelium with compromised barrier function.

Alternatively, the increased biosynthesis of cysteine (a precursor of glutathione) and of glutathione transport modules may speak to the microbiome's response to the oxidative stress (high levels of reactive oxygen and nitrogen species) of the inflamed IBD gut [76]. In support of this concept, we found that riboflavin metabolism, which is required to convert glutathione between its oxidized and reduced forms, is increased in iCD. Furthermore, the pentose phosphate pathway, which produces the NADPH also required for glutathione reduction, is increased as well. Recent studies have shown that redox stress allows *Salmonella *to use ethanolamine as a carbon source [80] and allows enterohemorrhagic *E. coli *to use it as a nitrogen source [81], thus conferring a competitive advantage to these microbes. This raises the interesting possibility that *E. coli *or related species in IBD may be highly represented because they gain a competitive advantage from oxidative stress and are better able to compensate for it with glutathione production.

In both UC and CD, there were decreases in the biosynthesis of lysine, arginine, and histidine in favor of transport in both UC and CD; a further decrease in tryptophan metabolism was associated with iCD. The data showed additional broad decreases in many essential processes, such as cobalamin synthesis, purine and pyrimidine biosythesis, lipid catabolism, and phospholipid metabolism, as well as marked increases in transport. This overall decrease in abundance of genes for amino acid and nucleotide biosynthesis bears striking resemblance to the lifestyle of highly symbiotic bacteria that are intrinsically auxotropic and also of some pathobionts (Figure 6). One such example are segmented filamentous bacteria (SFB), a symbiont that belongs to the *Candidadatus *Arthromitis, a sub-group of clade I *(sensu stricto*) Clostridia. A recently sequenced SFB genome lacked genes for nucleotide biosynthesis as well as nearly all vitamins and amino acids [82,83]. SFB are often abundant in the rodent terminal ileum and are responsible for the maturation of Th17 cells [84], which play an important role in CD-associated inflammation [85]. To date, neither SFB nor phylogenetically related sequences have been observed in humans [82,86]; this was also true in our data (zero 16S sequences with > 90% identity to X77814 SFB). However, a functional trend similar to SFB was observed in these IBD community metagenomes, as biosynthetic mechanisms throughout central carbon metabolism, amino acid biosynthesis, and nucleotide maintenance were all reduced (Figures 4 and 5), hinting that humans may host functional equivalents of SFB-like pathobionts that increase in IBD but are not phylogenetically close to *Candidatus *Arthromitis. Host tissue destruction, either inflammation-mediated or bacterially mediated, would provide a ready nutrient source (Figure 6).

## Conclusions

The data presented here show that IBD and iCD in particular are associated with a dysbiosis characterized by changes in Firmicutes and Proteobacteria phyla. Environmental factors and, notably, treatments were also associated with independent changes in the GI microbiome; these must be taken into account during future studies of the microbiota in IBD. These perturbations in bacterial composition, although modest, were associated with major perturbations of GI microbiome function, which revolved around metabolism in the presence of oxidative stress and perturbed nutrient availability during tissue damage. Further studies, particularly including transcriptomic, proteomic, or metabolomic characterization, longitudinal data, and dietary metadata, will be needed to additionally define the consequences of the IBD-associated microbiome dysfunction on the host and the specific mechanisms by which they are carried out or regulated by the microbiota.

## Materials and methods

### The OSCCAR and PRISM cohorts

The Ocean State Crohn's and Colitis Area Registry (OSCCAR) is a state-based, prospective inception cohort of IBD patients that was designed to study the epidemiology of IBD, to determine the incidence of IBD in Rhode Island, and to extrapolate these rates to the general population of the United States. The diverse population of over 1 million, limited geographic range, and well-circumscribed gastroenterology community of Rhode Island were ideal circumstances for establishing a prospective inception cohort of IBD patients. All but one of the 98 gastroenterologists/colorectal surgeons in Rhode Island agreed to refer patients to OSCCAR, and 11 gastroenterologists practicing in Massachusetts just over the Rhode Island border also agreed to refer their newly diagnosed IBD patients who resided in Rhode Island. Enrollment began 1 January 2008. All Rhode Island residents with a newly confirmed diagnosis of CD, UC, or indeterminate colitis were eligible for inclusion (within 12 months from diagnosis). Ethnic background of the subjects was not available for consideration in the analysis, and indeterminate colitis patients were analyzed only for other metadata and not for IBD diagnosis. Diagnosis of CD, UC, or indeterminate colitis was made by endoscopic, pathologic, or radiographic findings according to the criteria of the National Institute of Diabetes and Digestive and Kidney Diseases (NIDDK) IBD Genetics Consortium. OSCCAR research protocols were reviewed and approved by three institutional review boards (Lifespan (#0214-07), the Partners Human Research Committee (#2007-P-001705), and the Program for the Protection of Human Subjects/Mount Sinai School of Medicine (#11-01479)), and all experiments adhered to the regulations of these review boards. Informed consent and HIPAA (Health Insurance Portability and Accountability Act) authorization were obtained from each subject prior to study participation. Individuals diagnosed with IBD prior to the study start date, pregnant women, those unwilling to provide informed consent for study participation, and those who were prisoners at the time of diagnosis were not permitted to enroll.

The Prospective Registry in IBD Study at MGH (PRISM) is a referral center-based, prospective cohort of IBD patients. Enrollment began 1 January 2005. Patients aged 18 years and older with a diagnosis of CD or UC based upon standard endoscopic, radiographic, and histologic criteria were eligible to participate. Controls consisted of healthy patients aged 18 years and older, from whom biopsies were obtained during colonoscopies performed for screening purposes.

Patients were excluded from the healthy volunteer group for current acute illness, if awaiting transplant, or if chronically ill (for example, renal failure, diabetes, congestive heart failure). During routine colonoscopies, subjects were offered the opportunity to donate biopsy samples. After sampling, intestinal biopsies were stored in 5% glycerol at -80°C until DNA extraction. Stool samples were kept at 4°C for less than 24 h before storage at -80°C until DNA extraction. PRISM research protocols were reviewed and approved by the Partners Human Research Committee (#2004-P-001067), and all experiments adhered to the regulations of this review board.

### DNA extractions

DNA from stool and biopsy samples was extracted using the QIAamp DNA Stool Mini Kit (Qiagen, Inc., Valencia, CA, USA) according to manufacturer's instructions and as described previously [87]. The manufacturer's protocol was altered to accommodate larger stool volumes and to improve homogenization using bead-beating at several steps: a) a minimum of 2 ml of Buffer ASL and 300 mg of stool was used in the protocol; b) a ratio of 700 μl of Buffer ASL per 100 mg of stool weight was used for larger volumes using no more than 1,500 mg of stool and 10.5 ml of Buffer ASL; c) following the addition of Buffer ASL to each sample (step 2), 0.70 mm Garnet Beads (MO BIO Laboratories, Inc., Carlsbad, CA, USA) were added to the suspension and vortexed for 10 seconds; d) a second bead-beating was performed following the heating of the suspension (step 3) in 0.1 mm Glass Bead Tubes (MO BIO Laboratories, Inc.), and vortexed for 10 minutes.

### Amplification and 454 sequencing of the 16S gene

The 16S gene dataset consists of 454 FLX Titanium sequences spanning the V3 to V5 variable regions. Detailed protocols used for 16S amplification and sequencing are available on the Human Microbiome Project Data Analysis and Coordination Center website [88]. In brief, genomic DNA was subjected to 16S amplifications using primers designed incorporating the FLX Titanium adapters and a sample barcode sequence, allowing directional sequencing covering variable regions V5 to partial V3 (primers: 357F 5'-CCTACGGGAGGCAGCAG-3' and 926R 5' CCGTCAATTCMTTTRAGT-3'). PCR mixtures (25 μl) contained 10 ng of template, 1× Easy A reaction buffer (Stratagene, La Jolla, CA, USA), 200 mM of each dNTP (Stratagene), 200 nM of each primer, and 1.25 U AccuPrime hifi cloning enzyme (Invitrogen, Carlsbad, CA, USA). The cycling conditions for the V3-V5 consisted of an initial denaturation of 95°C for 2 minutes, followed by 25 cycles of denaturation at 95°C for 40 s, annealing at 50°C for 30 s, extension at 72°C for 5 minutes and a final extension at 72°C for 7 minutes. Amplicons were confirmed on 1.2% Flash Gels (Lonza, Rockland, ME, USA), purified with AMPure XP DNA purification beads (Beckman Coulter, Danvers, MA, USA) according to the manufacturer, and eluted in 25 μl of 1× low TE buffer (pH 8.0). Amplicons were quantified on Agilent Bioanalyzer 2100 DNA 1000 chips (Agilent Technologies, Santa Clara, CA, USA) and pooled in equimolar concentration. Emulsion PCR and sequencing were performed according to the manufacturer's specifications.

### Processing sequencing samples

Sequences were processed in a data curation pipeline implemented in MOTHUR [89], which removed sequences from the analysis if they were less than 200 nucleotides or greater than 600 nucleotides, had a low read quality score (< 25), contained ambiguous characters, had a non-exact barcode match, or had more than 4 mismatches to the reverse primer sequences (926R). Remaining sequences were assigned to samples based on barcode matches, and barcode and primer sequences were then trimmed. Chimeric sequences were identified using the ChimeraSlayer [90] algorithm, and reads were classified with the MSU RDP classifier v2.2 [91] using the taxonomy maintained at the Ribosomal Database Project (RDP 10 database, version 6). Sequencing depth after processing averaged 2,860 (standard deviation 1,730) reads per sample.

### Metagenome inference from microbiome composition

To construct an approximate gene catalog for each sample community, we used the gene content of 1,119 KEGG reference genomes to infer the approximate gene content of our detected phylotypes. We first matched the FastTree GreenGenes (GG) phylogeny [92] annotated with these KEGG genomes' organisms against the RDP taxonomy used for phylotyping. Each clade in the RDP taxonomy was mapped to the clade within the GG phylogeny that maximized the Jaccard index of overlapping named descendant genomes. That is, each genus-level phylotype was assigned to the GG clade containing the most genomes from that genus and fewest from other genera. Higher-level clades continued this pattern using the Jaccard index as an optimality criterion. The gene contents for ancestral clades were then reconstructed across the GG tree, beginning with each reference genome (tree leaf) summarized as a vector of KEGG ortholog (KO) [70] copy numbers (0, 1, or multiple copies of the gene annotated within the genome). Gene contents of each parent GG clade *g *were calculated by averaging all descendant genomes' *h *KO vectors, with weight *w*(*g, h*) inversely exponential to phylogenetic distance:

$$\overset{⇀}{g}=\underset{h\in descendants\left(g\right)}{\sum }w\left(g,h\right)\overset{⇀}{h}/\underset{h\in descendants\left(g\right)}{\sum }w\left(g,h\right)$$

$$w\left(g,h\right)=2^{=dist\left(g,h\right)}$$

for GG tree nodes *g *and *h *separated by phylogenetic branch length *dist*(*g, h*) and annotated with KO genome vectors $\overset{⇀}{g}$ and $\overset{⇀}{h}$.

Using this vector representation of genomes, the abundance of an individual gene family (KO) *i *in a community due to the presence of a specific phylotype *g *is the product of the corresponding gene count $\overset{⇀}{g}\left[i\right]$ and the measured abundance of phylotype *g*. Therefore, the total relative abundance of each KO was estimated for each sample by adding the individual contributions of all phylotypes present in the sample. Using this method, we inferred the functional composition for each sampled community. The inference process's accuracy was validated by comparing inferred KO abundances in 16S datasets from the Human Microbiome Project with their metagenomically sequenced counterparts (Additional file 15).

### Metabolic pathway reconstruction

Inferred per-community gene (KO) abundances were subsequently reconstructed into microbial pathway relative abundances using HUMAnN, the Human Microbiome Project metabolic reconstruction pipeline [71]. KOs were grouped into pathways represented as gene sets using HUMAnN, which chooses pathways by maximum parsimony using MinPath [93] and computes each pathway's relative abundance as a smoothed average over all genes within it, taking into account outliers and gap filling. We ran HUMAnN three times to reconstruct three complementary types of pathways from these genes: small metabolic modules (using KEGG's conjunctive normal form logic), large metabolic pathways, and Gene Ontology terms (using annotation-to-KO mappings from nine well-characterized KEGG microbes: ban, cje, cpe, eco, nse, pae, sce, son, and vch). For each of these three types of pathway, HUMAnN input the inferred relative abundances of all genes in each sample, and output the relative abundances of pathways within the sample. Subsequent analysis handled these sample-by-pathway relative abundances in the same manner as sample-by-clade microbial abundances.

### Significant associations of microbial clades and pathways with sample metadata

Inverse Simpson diversity, Chao1 richness (using the R fossil package), and Pielou evenness were calculated for clade abundance, KEGG pathway and module abundance, and Gene Ontology term abundance [94-97]. Next, data were pre-processed for quality control before modeling. Clinical metadata were removed when more than 10% of data were missing, or when they did not vary in value over the available samples. Clades, pathways, and features of very low abundance (< 0.001 in ≥ 90% of samples) and feature outliers outside of the lower or upper outer fence (3× interquartile range) were removed. Missing data were imputed for significance testing with the mean abundance of the sample; missing factor metadata were imputed with a 'NA' factor level using the na.gam.replace function from the R package [98]. Unless stated otherwise, all subsequent analyses and calculations were performed using these processed data. After processing, 228 and 231 samples passed quality control for clade abundance and functional abundance analyses, respectively.

Finally, clades and functions were tested for statistically significant associations with clinical metadata of interest by using a novel multivariate algorithm. Each clade (excluding ecological measures) was normalized with a variance-stabilizing arcsine square-root transformation and evaluated with a general linear model (in R using the glm package). Model selection for sparse data was performed per clade using boosting (gbm package [99]). A multivariate linear model associating all available metadata with each clade independently was boosted, and any metadata selected in at least 1% of these iterations was finally tested for significance in a standard generalized linear model. This composite model was thus of the form:

$$\text{arcsin}\left(\sqrt{y_{i}}\right))=\beta_{0}+\underset{p}{\sum }\beta_{p}X_{i,p}+\varepsilon_{i},i=1,...,n$$

where *p *are the clinical metadata selected from boosting.

Within each metadatum/clade association independently, multiple comparisons over factor levels were adjusted using a Bonferonni correction; multiple hypothesis tests over all clades and metadata were adjusted to produce a final Benjamini and Hochberg false discovery rate [100]. Unless otherwise indicated, significant association was considered below a q-value threshold of 0.25; the KEGG pathway sulfur metabolism (ko00920) had an average q-value of 0.26 for association with Crohn's disease. Multiple factor analysis was performed to visualize the relationships within heterogeneous factor data as well as with a select group of taxa found to be significantly associated with metadata (using the FactoMineR R package [101]). Total abundances and significant associations between metadata, taxa, and functions are listed in Additional files 1 and 11.

### Sequence alignment for segmented filamentous bacteria

To determine whether SFB were present in samples, three sequences of SFB (X80834, X87244, and X77814) from three species (chicken, rat, and mouse) were aligned by blastn, using both a 20 and 15 seed word. No sequences were found with ≥ 95% identity over an alignment length of at least 100 nucleotides. The average sequence length from the study was 435 nucleotides.

### Shotgun metagenomic sequencing

To provide internal validation of inferred microbial community gene and pathway compositions, stool DNA from seven healthy controls and four CD patients was subjected to metagenomic shotgun sequencing. Libraries were constructed with the Illumina Nextera XT kit and sequenced on an Illumina MiSeq using 2 × 150 bp paired-end sequencing according to the manufacturer's instructions. This resulted in sequencing depths ranging from 3.9 to 270 meganucleotides, average 119 meganucleotides, from which microbial community function was determined with HUMAnN [71] as described above.

### Sequence accession numbers and availability

Sequences generated in this study are publicly available (NCBI BioProject ID numbers 82111 and 175224).

## Abbreviations

CD: Crohn's disease; GG: GreenGenes; GI: gastrointestinal; IBD: inflammatory bowel disease; iCD: ileal Crohn's disease; KEGG: Kyoto Encyclopedia of Genes and Genomes; KO: KEGG ortholog; OSCCAR: Ocean State Crohn's and Colitis Area Registry; PRISM: Prospective Registry in IBD Study at MGH; RDP: Ribosomal Database Project; SCFA: short-chain fatty acid; SFB: segmented filamentous bacteria; TLR: toll-like receptor; UC: ulcerative colitis.

## Competing interests

The authors declare that they have no competing interests.

## Authors' contributions

DG, BES, RJX, and CH conceived and designed the study. DG, DVW, and XCM generated sequencing data. HS, TLT, XCM, and CH analyzed data. TLT, DG, and JAR performed computational analysis. HS, XCM, SBS, AB, JK, BES, RJX, and CH interpreted data. DG, KLD, DVW, SAS, NL, AB, BES, and RJX coordinated sample analysis. HS, TLT, XCM, and CH drafted the manuscript. All authors have read and approved the manuscript for publication.

## Supplementary Material

Additional file 1 **Taxa significantly associated with IBD status or subject metadata using a boosted general linear model**. A multivariate analysis was performed to associate each microbial clade with a sparse selection of disease status and clinical metadata (selected through boosting; see Materials and methods). All clades and metadata in these associations are given with nominal *P*-values from the multivariate linear model and with Benjamini and Hochberg (BH) corrected false discovery rate (q-values) up to a threshold of 0.25. In this and all other supplemental tables, blank spaces indicate values that were not significant but are shown for comparison with related significant data. Click here for file

Additional file 2 **Effects of biogeography on gut microbiome composition differentiates stool and biopsy communities**. The composition of phyla stratified by biopsy location or fecal sample origin mainly differentiates stool and biopsy communities. Sample count per location is indicated in parentheses. Biopsy locations (above) do not substantially differ in composition, while biopsies compared to stool (below) differ significantly in all phyla. Click here for file

Additional file 3 **Univariate analysis of associations between microbial composition and biopsy location**. A univariate analysis for associations between taxa and biopsy sites was conducted using LEfSe [102] considering the six regions annotated for these samples: 1) terminal ileum (TI), 2) cecum, 3) left colon, 4) transverse colon, 5) right colon, and 6) sigmoid colon and rectum. **(a) **Relatively few clades were strongly associated with biopsy locations, and these tended to mirror expected intestinal pH and the clades described here as particularly affected by disease-linked inflammation. **(b-g) **Abundant major clades, including the Firmicutes (b), showed extremely modest variations with intestinal region, driven by specific members depleted in low-pH regions, including *Roseburia *(c) (high in the left and sigmoid colon), Ruminococcaceae (d), and to a lesser degree *Alistipes *(e). Clades enriched in low pH regions included *Fusobacterium *(f) (high in TI and right colon) and Enterobacteriales (g) (particularly in TI). Click here for file

Additional file 4 **Locations of patient biopsies**. Distribution of biopsy samples available for this study as classified by the OSCCAR and PRISM cohort collection protocol. Click here for file

Additional file 5 **Univariate analysis of associations between microbial composition and gender**. A univariate test for associations of subject gender with microbial clades was conducted using LEfSe [102], resulting in few and weak associations concordant with previous studies [5]. Here, *Clostridium *and the Streptococcaceae were weakly associated with gender at *P *< 0.05, but did not remain significant at *P *< 0.1. Click here for file

Additional file 6 ***Bifidobacterium *genus abundance decreases significantly with age**. The association of *Bifidobacterium *abundance with disease status and clinical metadata (including age) was determined to be significant in these data using a sparse general linear model. Clade abundances were transformed with the arcsine square-root transformation for proportional data (y-axis). Size of effect, standard deviation, *P*-value (p) and Benjamini and Hochberg false discovery rate (q) are shown in parentheses, and the line of best fit in green. Click here for file

Additional file 7 ***Escherichia/Shigella *abundance is significantly decreased in mesalamine-treated subjects**. The association of these genera (indistinguishable by 16S rRNA gene sequencing) with disease status and clinical metadata (including mesalamine treatment) was determined to be significant using a sparse general linear model (see Materials and methods). Clade abundances were transformed with the arcsine square root transformation for proportional data and are plotted along the y-axis as two notched box plots (samples without and with mesalamine use). Size of effect, standard deviation, *P*-value (p) and q-value (q) are shown in parentheses. Click here for file

Additional file 8 **Stratification of clades associated with IBD status by sample biogeography**. Fifteen microbial clades were significantly associated specifically with IBD status (q < 0.25) using a multivariate linear model incorporating clinical metadata (see Materials and methods). Although this model putatively asserts that this association holds regardless of sample origin (biopsy or stool), we verified this by stratifying each clade's abundance by sample type, stool (1) or biopsy (0). Green coloring indicates that a clade's abundance was significantly reduced in IBD using the full model, red increased. These trends are uniformly preserved after explicit stratification by stool versus biopsy sample origins. Click here for file

Additional file 9 **Univariate associations of microbial composition with biopsy location**. Results of a LEfSe analysis of the six location categories available for biopsies in this study, excluding two anatostamosis samples. Click here for file

Additional file 10 **Covariation of microbial community function in IBD with treatment, environment, biometrics, and disease subtype**. Fecal and biopsy samples from 231 IBD patients and healthy controls are plotted as squares (iCD) or circles and colored by disease status. Axes show the first two components of overall variation as determined by multiple factor analysis (see Materials and methods). Clinical and environmental covariates are shown in bold, while individual microbial functions (Gene Ontology terms) are italicized. Covariation patterns are similar to those determined using microbial abundance (Figure 1). Click here for file

Additional file 11 **KEGG pathways significantly associated with IBD status or subject metadata using a boosted general linear model**. A multivariate analysis was performed to associate each pathway with a sparse selection of disease status and clinical metadata (selected through boosting; see Materials and methods). All pathways and metadata in these associations are given with nominal *P*-values from the multivariate linear model and with Benjamini and Hochberg (BH) corrected false discovery rate (q-values) up to a threshold of 0.25. Click here for file

Additional file 12 **KEGG metabolic modules significantly associated with IBD status or subject metadata using a boosted general linear model**. A multivariate analysis was performed to associate each metabolic module with a sparse selection of disease status and clinical metadata (selected through boosting; see Materials and methods). Each module and metadata in these associations is given with nominal p-values from the multivariate linear model and with Benjamini and Hochberg (BH) corrected false discovery rate (q-values) up to a threshold of 0.25. Click here for file

Additional file 13 **Gene Ontology terms significantly associated with IBD status or subject metadata using a boosted general linear model**. A multivariate analysis was performed to associate each Gene Ontology term with a sparse selection of disease status and clinical metadata (selected through boosting; see Materials and methods). Each term and metadata in these associations is given with nominal *P*-values from the multivariate linear model and with Benjamini and Hochberg (BH) corrected false discovery rate (q-values) up to a threshold of 0.25. Click here for file

Additional file 14 **Shotgun metagenomic sequencing validates predicted microbial metabolic trends in a subset of healthy and CD microbiomes**. A subset of 11 stool samples for which microbial DNA was available were subjected to shallow metagenomic sequencing using the MiSeq platform (150-nucleotide paired-end reads) averaging 119 meganucleotides per sample. **(a) **Of the seven microbial metabolic modules highlighted in Figure 5, six retained the same over- or under-abundance trend predicted from 16S sequencing in this subset, with the seventh (cobalamin biosynthesis) falling below the limit of detection. **(b) **Six additional metabolic modules of interest with significant differences in the full IBD dataset retained the trend expected with CD in this subset, including depletion of glycolysis processes and enrichment for bacterial secretion systems. Click here for file

Additional file 15 **Correlation of microbial gene families estimated from 16S gene pyrosequencing and whole-genome shotgun sequencing data**. Ancestral state reconstruction was used to infer metagenomes using 16S gene pyrosequencing of samples from multiple body sites from the Human Microbiome Project (see Materials and methods). The relative abundance of KOs inferred from 16S sequencing and measured from paired whole-community genome sequencing samples were correlated (Spearman rank correlation) and plotted per body site. Each box plot shows the distribution of the correlation of relative KO abundance from 16S and whole-genome sequencing; specific sample-pair correlations are plotted as dots. Median correlation for Human Microbiome Project stool samples is 0.75 for an average *n *= 75 per body site. As each correlation is calculated over approximately 5,400 KOs, correlation values above 0.59 are significant at a Bonferroni-corrected *P *< 0.05. Click here for file
