# Supplementary figures and images for: Dysfunction of the intestinal microbiome in inflammatory bowel disease and treatment
Source: Genome Biol. 2012 Sep 26;13(9):R79. doi: 10.1186/gb-2012-13-9-r79 (PMC3506950; doi:10.1186/gb-2012-13-9-r79)

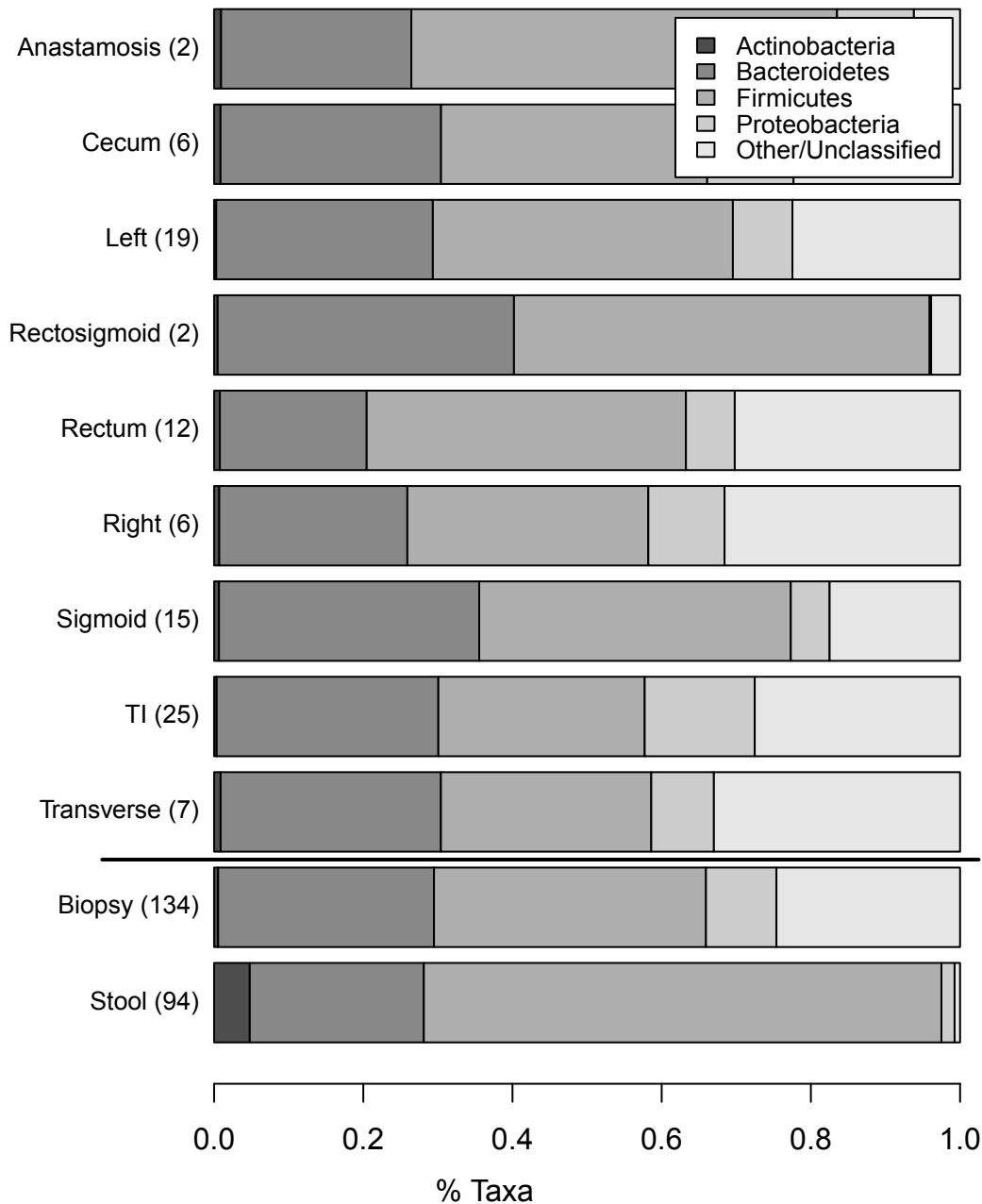

Supplement: Additional file 2 — Effects of biogeography on gut microbiome composition differentiates stool and biopsy communities. The composition of phyla stratified by biopsy location or fecal sample origin mainly differentiates stool and biopsy communities. Sample count per location is indicated in parentheses. Biopsy locations (above) do not substantially differ in composition, while biopsies compared to stool (below) differ significantly in all phyla. [file gb-2012-13-9-r79-S2.PDF]

Age (se -0.000929, sd 0.000267, p=0.000611, q=0.0198)

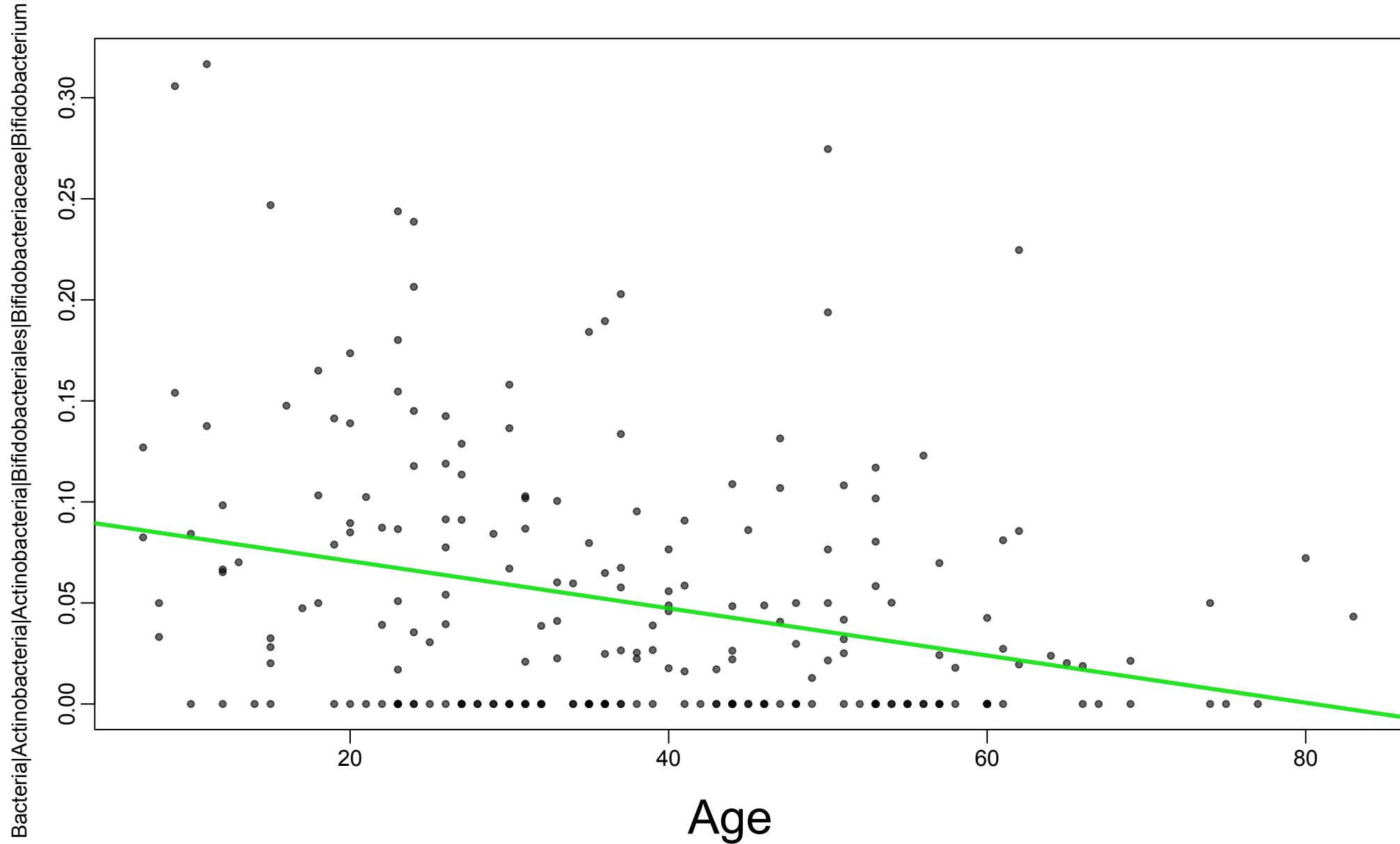

Supplement: Additional file 6 — Bifidobacterium genus abundance decreases significantly with age. The association of Bifidobacterium abundance with disease status and clinical metadata (including age) was determined to be significant in these data using a sparse general linear model. Clade abundances were transformed with the arcsine square-root transformation for proportional data (y-axis). Size of effect, standard deviation, P-value (p) and Benjamini and Hochberg false discovery rate (q) are shown in parentheses, and the line of best fit in green. [file gb-2012-13-9-r79-S6.PDF]

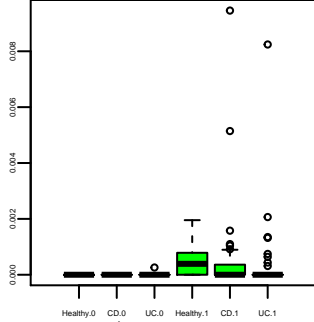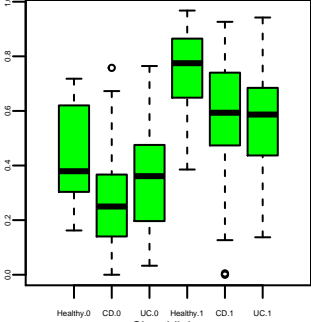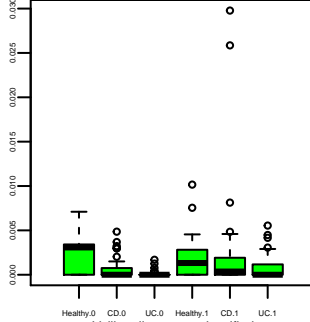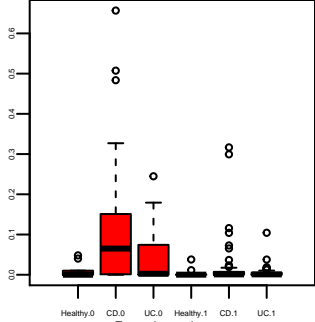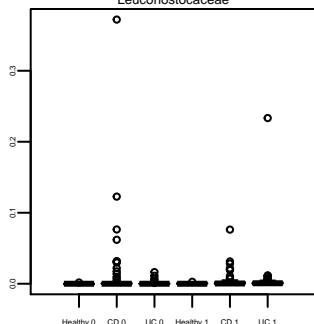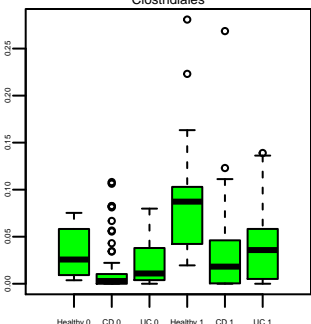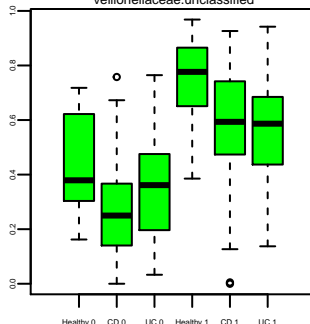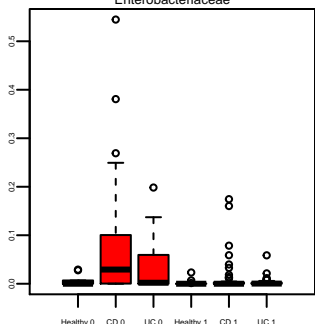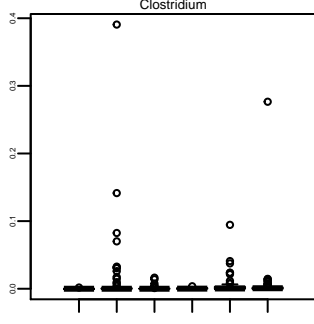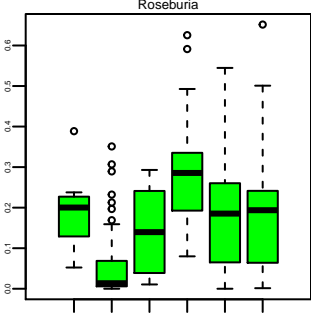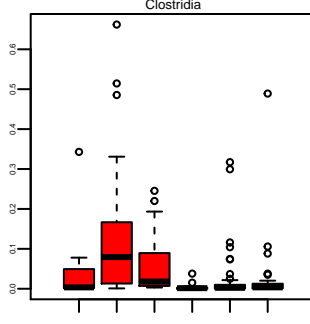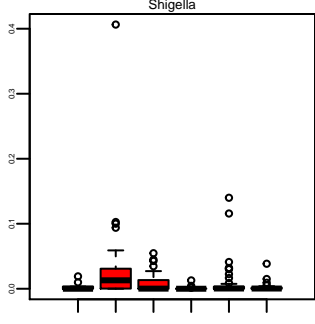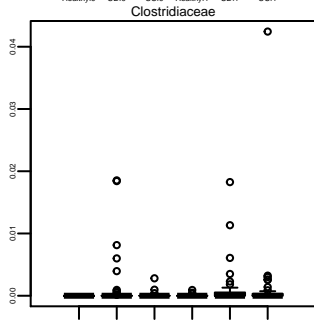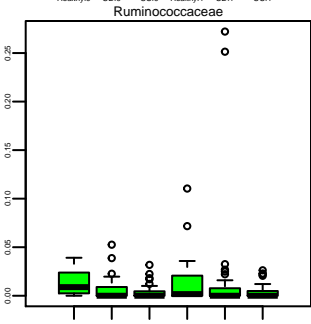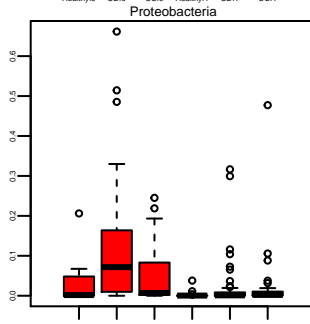

Supplement: Additional file 8 — Stratification of clades associated with IBD status by sample biogeography. Fifteen microbial clades were significantly associated specifically with IBD status (q < 0.25) using a multivariate linear model incorporating clinical metadata (see Materials and methods). Although this model putatively asserts that this association holds regardless of sample origin (biopsy or stool), we verified this by stratifying each clade's abundance by sample type, stool (1) or biopsy (0). Green coloring indicates that a clade's abundance was significantly reduced in IBD using the full model, red increased. These trends are uniformly preserved after explicit stratification by stool versus biopsy sample origins. [file gb-2012-13-9-r79-S8.PDF]

# Microbial metabolism (KEGG modules) differentially abundant in IBD

**A**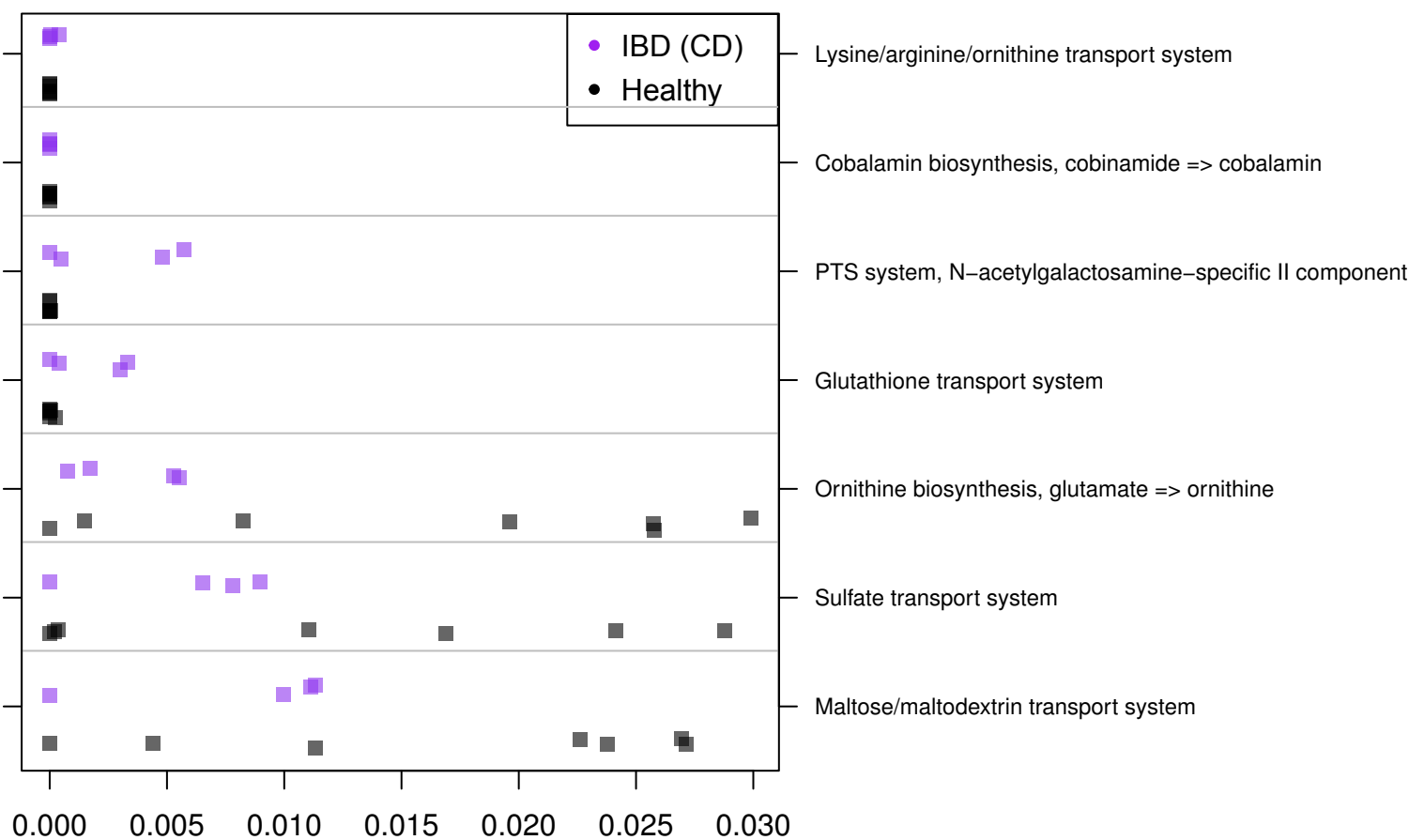**B**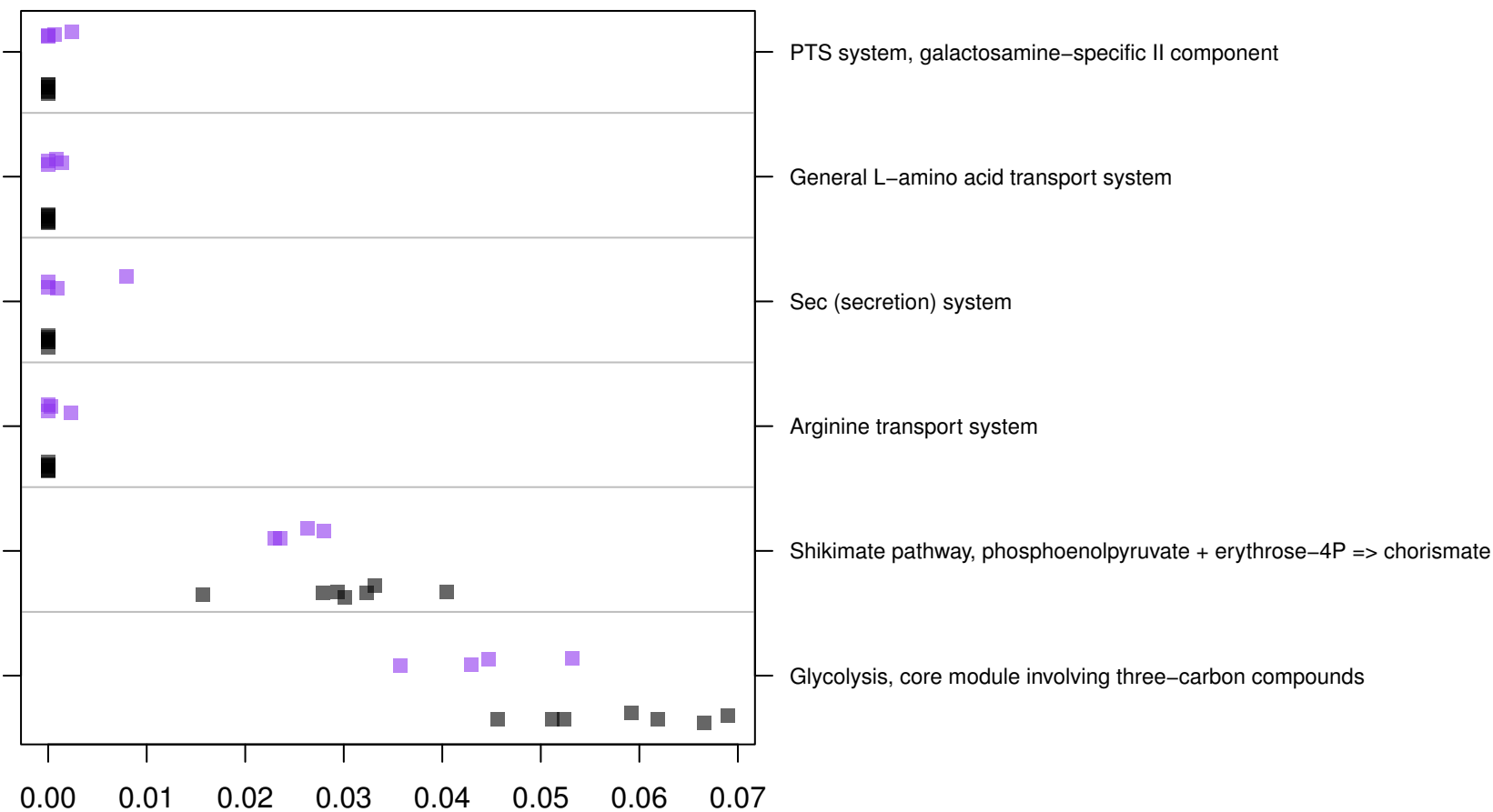

Relative Abundance

Supplement: Additional file 14 — Shotgun metagenomic sequencing validates predicted microbial metabolic trends in a subset of healthy and CD microbiomes. A subset of 11 stool samples for which microbial DNA was available were subjected to shallow metagenomic sequencing using the MiSeq platform (150-nucleotide paired-end reads) averaging 119 meganucleotides per sample. (a) Of the seven microbial metabolic modules highlighted in Figure 5, six retained the same over- or under-abundance trend predicted from 16S sequencing in this subset, with the seventh (cobalamin biosynthesis) falling below the limit of detection. (b) Six additional metabolic modules of interest with significant differences in the full IBD dataset retained the trend expected with CD in this subset, including depletion of glycolysis processes and enrichment for bacterial secretion systems. [file gb-2012-13-9-r79-S14.PDF]

# Correlation of microbial gene families estimated from 16S and WGS sequence data

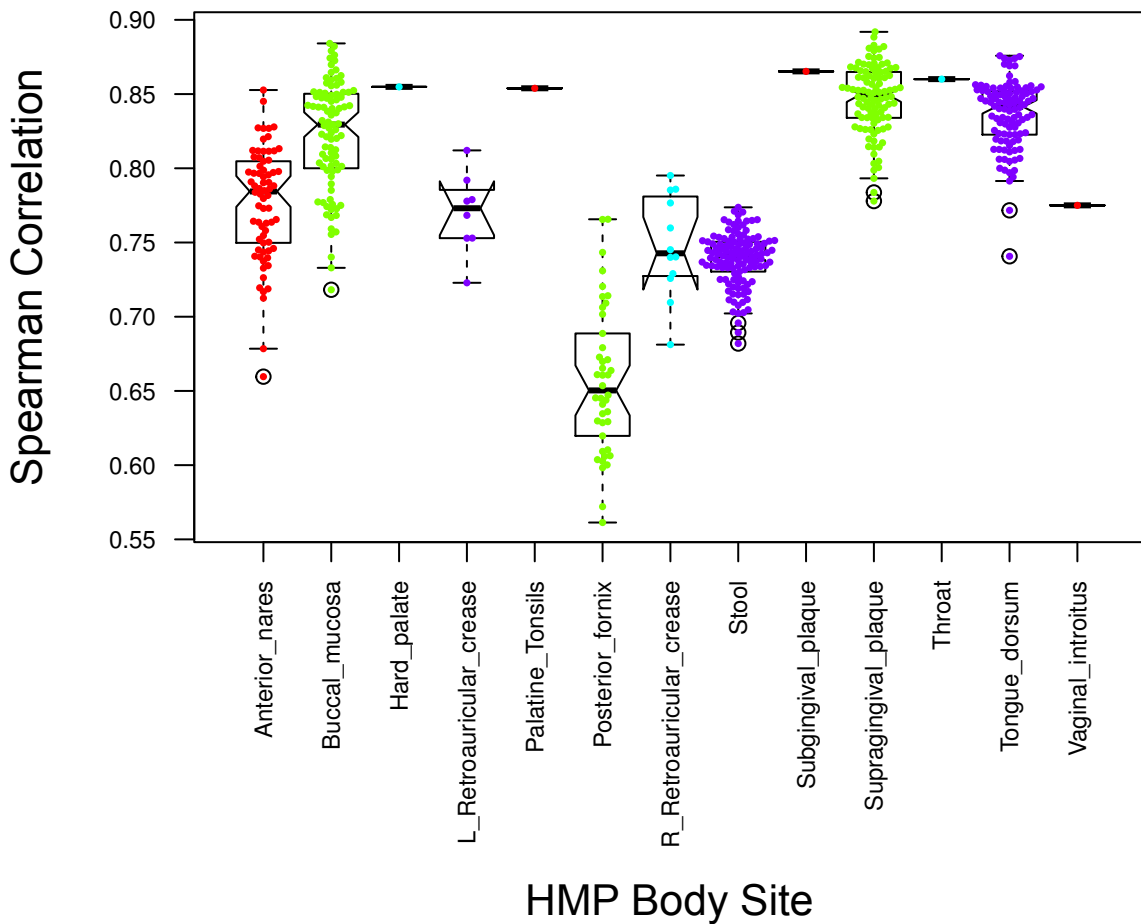

Supplement: Additional file 15 — Correlation of microbial gene families estimated from 16S gene pyrosequencing and whole-genome shotgun sequencing data. Ancestral state reconstruction was used to infer metagenomes using 16S gene pyrosequencing of samples from multiple body sites from the Human Microbiome Project (see Materials and methods). The relative abundance of KOs inferred from 16S sequencing and measured from paired whole-community genome sequencing samples were correlated (Spearman rank correlation) and plotted per body site. Each box plot shows the distribution of the correlation of relative KO abundance from 16S and whole-genome sequencing; specific sample-pair correlations are plotted as dots. Median correlation for Human Microbiome Project stool samples is 0.75 for an average n = 75 per body site. As each correlation is calculated over approximately 5,400 KOs, correlation values above 0.59 are significant at a Bonferroni-corrected P < 0.05. [file gb-2012-13-9-r79-S15.PDF]
