# Supplementary material for: Dysfunction of the intestinal microbiome in inflammatory bowel disease and treatment
Source: Genome Biol. 2012 Sep 26;13(9):R79. doi: 10.1186/gb-2012-13-9-r79 (PMC3506950; doi:10.1186/gb-2012-13-9-r79)
Supplement: Additional file 3 — Univariate analysis of associations between microbial composition and biopsy location. A univariate analysis for associations between taxa and biopsy sites was conducted using LEfSe [102] considering the six regions annotated for these samples: 1) terminal ileum (TI), 2) cecum, 3) left colon, 4) transverse colon, 5) right colon, and 6) sigmoid colon and rectum. (a) Relatively few clades were strongly associated with biopsy locations, and these tended to mirror expected intestinal pH and the clades described here as particularly affected by disease-linked inflammation. (b-g) Abundant major clades, including the Firmicutes (b), showed extremely modest variations with intestinal region, driven by specific members depleted in low-pH regions, including Roseburia (c) (high in the left and sigmoid colon), Ruminococcaceae (d), and to a lesser degree Alistipes (e). Clades enriched in low pH regions included Fusobacterium (f) (high in TI and right colon) and Enterobacteriales (g) (particularly in TI). [file gb-2012-13-9-r79-S3.PDF]

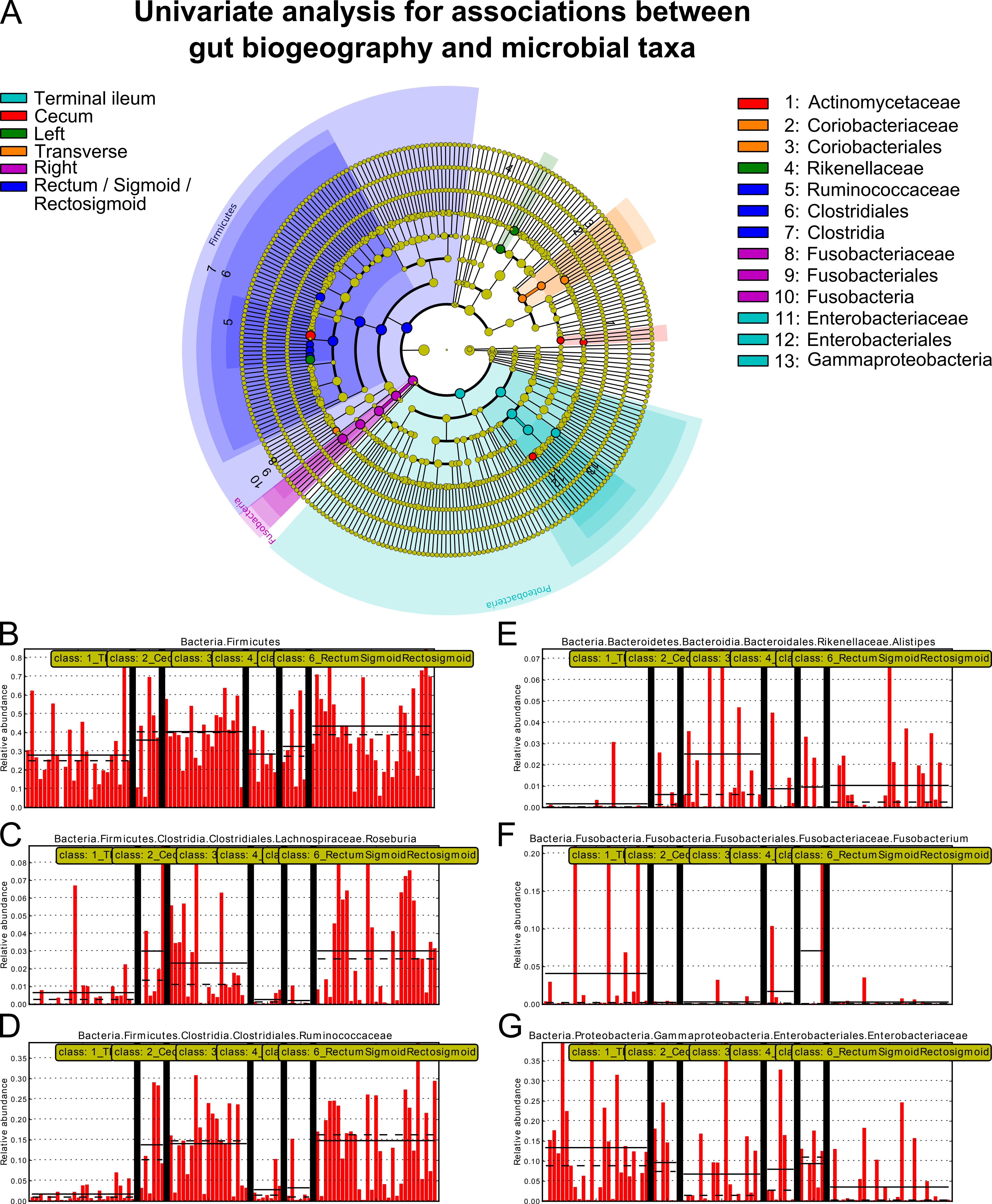

A
Univariate analysis for associations between gut biogeography and microbial taxa
Terminal ileum
Cecum
Left
Transverse
Right
Rectum / Sigmoid / Rectosigmoid
1: Actinomycetaceae
2: Coriobacteriaceae
3: Coriobacteriales
4: Rikenellaceae
5: Ruminococcaceae
6: Clostridiales
7: Clostridia
8: Fusobacteriaceae
9: Fusobacteriales
10: Fusobacteria
11: Enterobacteriaceae
12: Enterobacteriales
13: Gammaproteobacteria
Firmicutes
Fusobacteria
Proteobacteria
B
Bacteria.Firmicutes
C
Bacteria.Firmicutes.Clostridia.Clostridiales.Lachnospiraceae.Roseburia
D
Bacteria.Firmicutes.Clostridia.Clostridiales.Ruminococcaceae
E
Bacteria.Bacteroidetes.Bacteroidia.Bacteroidales.Rikenellaceae.Alistipes
F
Bacteria.Fusobacteria.Fusobacteria.Fusobacteriales.Fusobacteriaceae.Fusobacterium
G
Bacteria.Proteobacteria.Gammaproteobacteria.Enterobacteriales.Enterobacteriaceae
class: 6_RectumSigmoidRectosigmoid
Relative abundance
