# Supplementary material for: Dysfunction of the intestinal microbiome in inflammatory bowel disease and treatment
Source: Genome Biol. 2012 Sep 26;13(9):R79. doi: 10.1186/gb-2012-13-9-r79 (PMC3506950; doi:10.1186/gb-2012-13-9-r79)
Supplement: Additional file 4 — Locations of patient biopsies. Distribution of biopsy samples available for this study as classified by the OSCCAR and PRISM cohort collection protocol. [file gb-2012-13-9-r79-S4.DOCX]

|  | CD | UC | HS | Indeterminate |
| --- | --- | --- | --- | --- |
| **n** | 121 | 75 | 27 | 8 |
| **Biopsy Location :** |  |  |  |  |
| Anatostamosis % (n) | 3.3% (2) | 0% (0) | 0% (0) | 0% (0) |
| Cecum % (n) | 3.3% (2) | 14.8% (4) | 0% (0) | 0% (0) |
| Left % (n) | 13.6% (8) | 29.6% (8) | 33.3% (3) | 0% (0) |
| Rectosigmoid % (n) | 0% (0) | 0% (0) | 22.2% (2) | 0% (0) |
| Rectum % (n) | 8.5% (5) | 25.9% (7) | 0% (0) | 0% (0) |
| Right % (n) | 10.1% (6) | 0% (0) | 0% (0) | 0% (0) |
| Sigmoid % (n) | 6.8% (4) | 25.9% (7) | 44.4% (4) | 0% (0) |
| Terminal ileum % (n) | 42.3% (25) | 0% (0) | 0% (0) | 0% (0) |
| Transverse % (n) | 10.1% (6) | 3.7% (1) | 0% (0) | 0% (0) |
| Unknown % (n) | 1.7% (1) | 0% (0) | 0% (0) | 0% (0) |
