# Supplementary material for: Dysfunction of the intestinal microbiome in inflammatory bowel disease and treatment
Source: Genome Biol. 2012 Sep 26;13(9):R79. doi: 10.1186/gb-2012-13-9-r79 (PMC3506950; doi:10.1186/gb-2012-13-9-r79)

Univariate analysis for associations between gender and microbial taxa

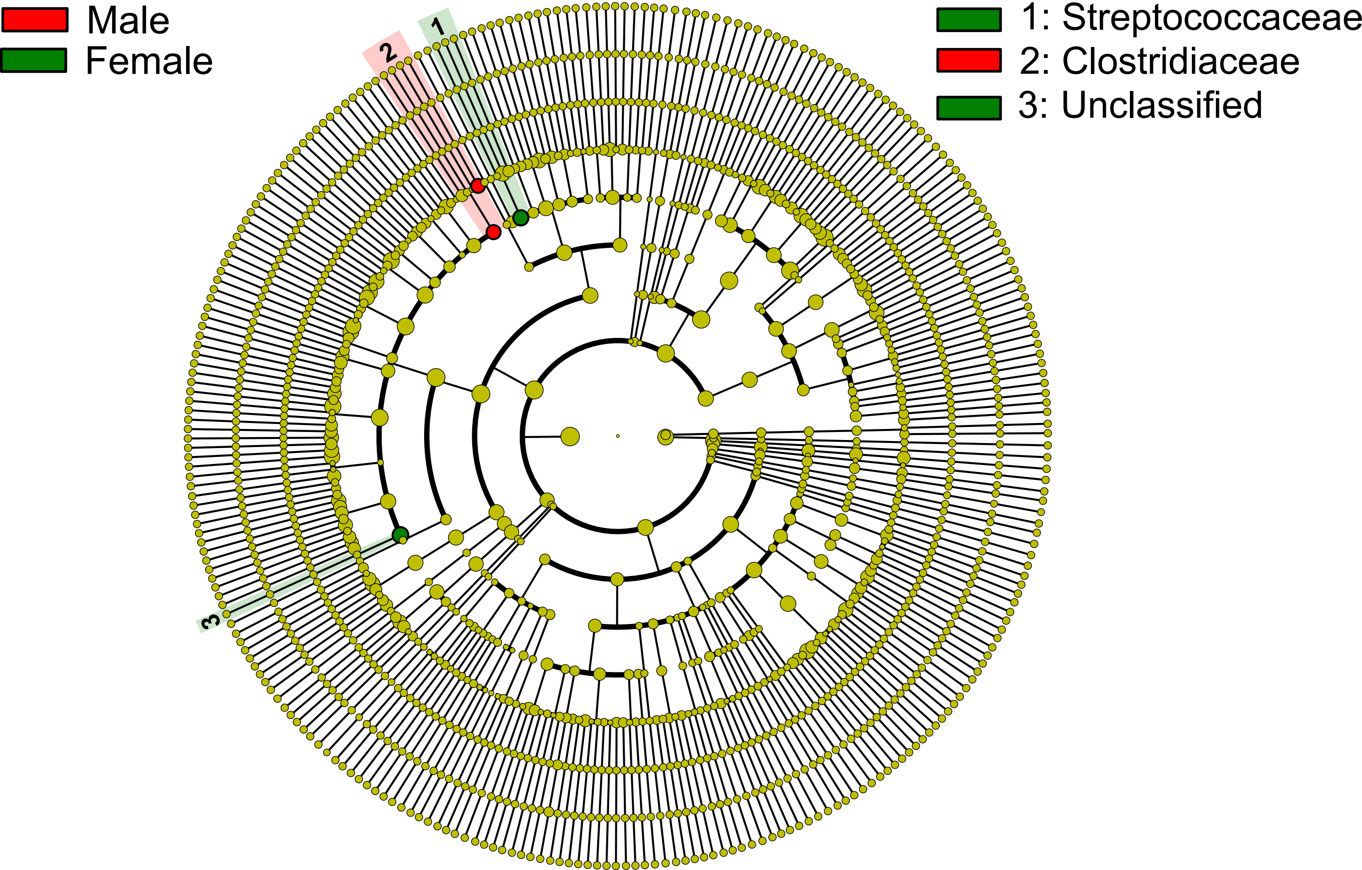

Supplement: Additional file 5 — Univariate analysis of associations between microbial composition and gender. A univariate test for associations of subject gender with microbial clades was conducted using LEfSe [102], resulting in few and weak associations concordant with previous studies [5]. Here, Clostridium and the Streptococcaceae were weakly associated with gender at P < 0.05, but did not remain significant at P < 0.1. [file gb-2012-13-9-r79-S5.PDF]
