# Supplementary material for: Dysfunction of the intestinal microbiome in inflammatory bowel disease and treatment
Source: Genome Biol. 2012 Sep 26;13(9):R79. doi: 10.1186/gb-2012-13-9-r79 (PMC3506950; doi:10.1186/gb-2012-13-9-r79)
Supplement: Additional file 7 — Escherichia/Shigella abundance is significantly decreased in mesalamine-treated subjects. The association of these genera (indistinguishable by 16S rRNA gene sequencing) with disease status and clinical metadata (including mesalamine treatment) was determined to be significant using a sparse general linear model (see Materials and methods). Clade abundances were transformed with the arcsine square root transformation for proportional data and are plotted along the y-axis as two notched box plots (samples without and with mesalamine use). Size of effect, standard deviation, P-value (p) and q-value (q) are shown in parentheses. [file gb-2012-13-9-r79-S7.PDF]

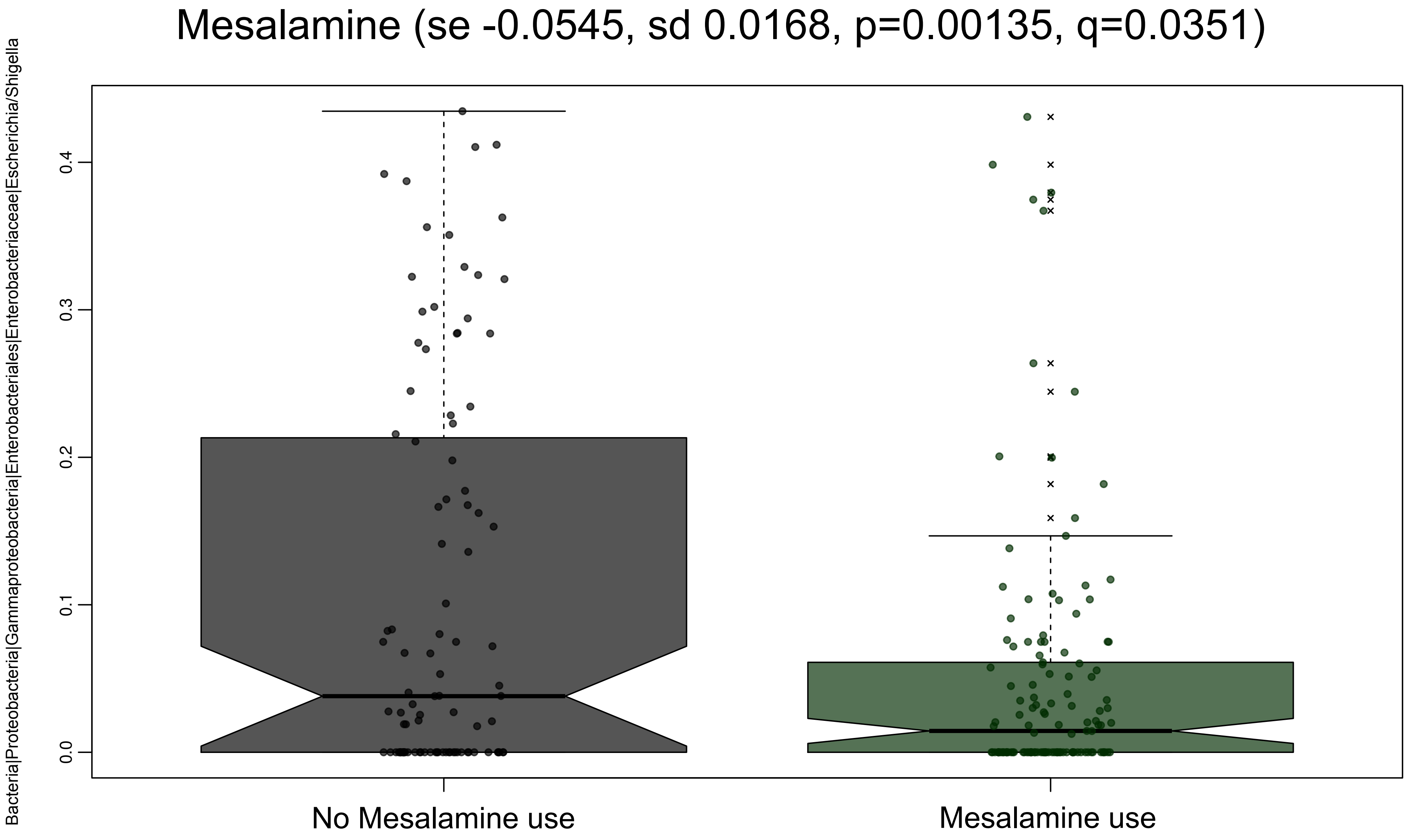
Mesalamine (se -0.0545, sd 0.0168, p=0.00135, q=0.0351)
Bacteria|Proteobacteria|Gammaproteobacteria|Enterobacteriales|Enterobacteriaceae|Escherichia/Shigella
0.0
0.1
0.2
0.3
0.4
No Mesalamine use
Mesalamine use
