# Supplementary material for: Dysfunction of the intestinal microbiome in inflammatory bowel disease and treatment
Source: Genome Biol. 2012 Sep 26;13(9):R79. doi: 10.1186/gb-2012-13-9-r79 (PMC3506950; doi:10.1186/gb-2012-13-9-r79)
Supplement: Additional file 10 — Covariation of microbial community function in IBD with treatment, environment, biometrics, and disease subtype. Fecal and biopsy samples from 231 IBD patients and healthy controls are plotted as squares (iCD) or circles and colored by disease status. Axes show the first two components of overall variation as determined by multiple factor analysis (see Materials and methods). Clinical and environmental covariates are shown in bold, while individual microbial functions (Gene Ontology terms) are italicized. Covariation patterns are similar to those determined using microbial abundance (Figure 1). [file gb-2012-13-9-r79-S10.PDF]

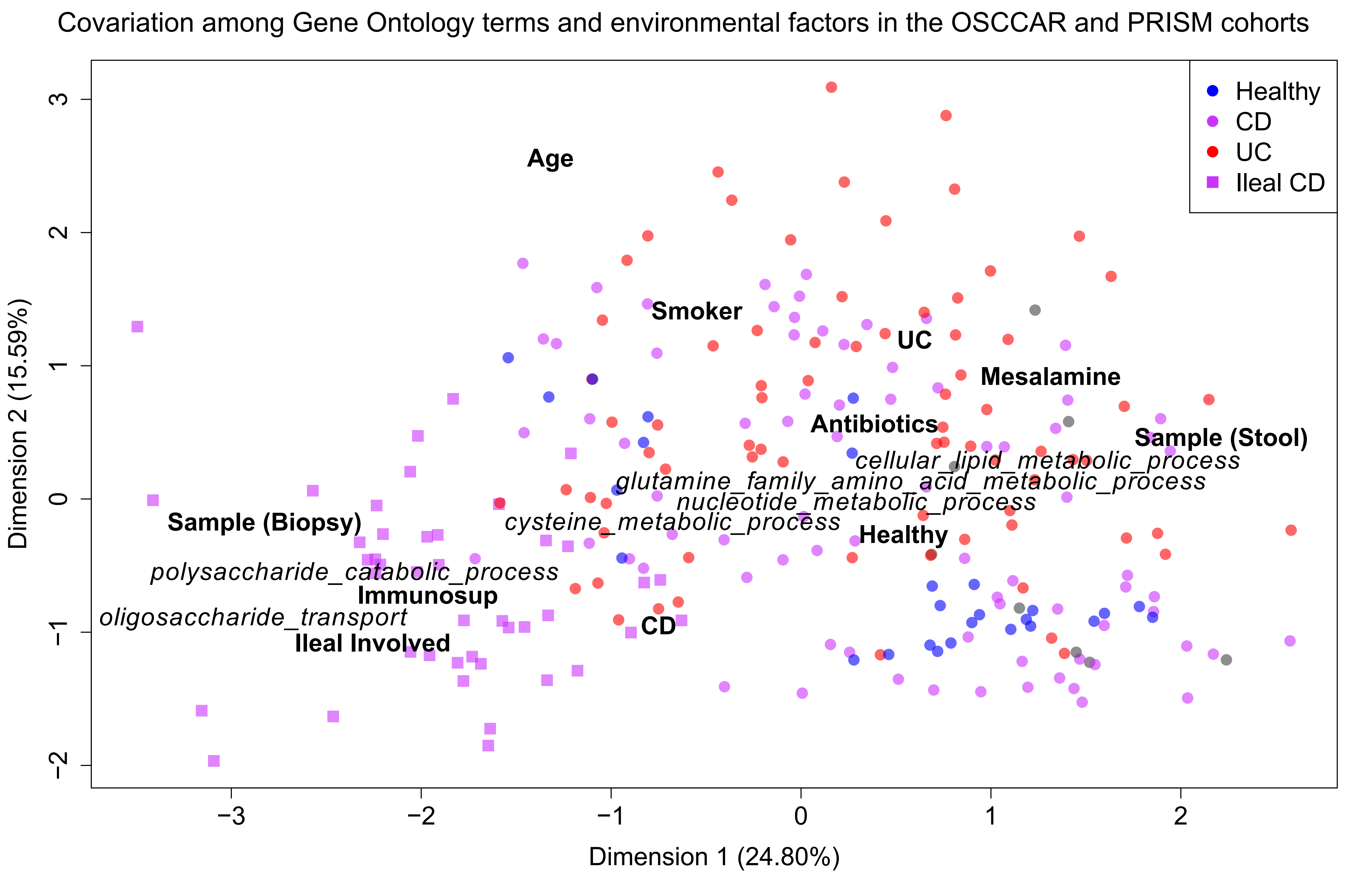

Covariation among Gene Ontology terms and environmental factors in the OSCCAR and PRISM cohorts
Healthy
CD
UC
Ileal CD
Age
Smoker
UC
Mesalamine
Antibiotics
Sample (Stool)
cellular_lipid_metabolic_process
glutamine_family_amino_acid_metabolic_process
nucleotide_metabolic_process
cysteine_metabolic_process
Sample (Biopsy)
Healthy
polysaccharide_catabolic_process
Immunosup
oligosaccharide_transport
CD
Ileal Involved
Dimension 2 (15.59%)
Dimension 1 (24.80%)
3
2
1
0
−1
−2
−3
−2
−1
0
1
2
